# Accurate identification of structural variations from cancer samples

**DOI:** 10.1093/bib/bbad520

**Published:** 2024-01-16

**Authors:** Le Li, Chenyang Hong, Jie Xu, Claire Yik-Lok Chung, Alden King-Yung Leung, Delbert Almerick T Boncan, Lixin Cheng, Kwok-Wai Lo, Paul B S Lai, John Wong, Jingying Zhou, Alfred Sze-Lok Cheng, Ting-Fung Chan, Feng Yue, Kevin Y Yip

**Affiliations:** Department of Computer Science and Engineering, The Chinese University of Hong Kong, Shatin, New Territories, Hong Kong; Department of Computer Science and Engineering, The Chinese University of Hong Kong, Shatin, New Territories, Hong Kong; Department of Biochemistry and Molecular Genetics, Feinberg School of Medicine, Northwestern University, Chicago, Illinois 60208, USA; School of Life Sciences and State Key Laboratory of Agrobiotechnology, The Chinese University of Hong Kong, Shatin, New Territories, Hong Kong; School of Life Sciences and State Key Laboratory of Agrobiotechnology, The Chinese University of Hong Kong, Shatin, New Territories, Hong Kong; School of Life Sciences and State Key Laboratory of Agrobiotechnology, The Chinese University of Hong Kong, Shatin, New Territories, Hong Kong; Department of Computer Science and Engineering, The Chinese University of Hong Kong, Shatin, New Territories, Hong Kong; Department of Anatomical and Cellular Pathology, The Chinese University of Hong Kong, Shatin, New Territories, Hong Kong; Department of Surgery, The Chinese University of Hong Kong, Shatin, New Territories, Hong Kong; Department of Surgery, The Chinese University of Hong Kong, Shatin, New Territories, Hong Kong; School of Biomedical Sciences, The Chinese University of Hong Kong, Shatin, New Territories, Hong Kong; School of Biomedical Sciences, The Chinese University of Hong Kong, Shatin, New Territories, Hong Kong; School of Life Sciences and State Key Laboratory of Agrobiotechnology, The Chinese University of Hong Kong, Shatin, New Territories, Hong Kong; Hong Kong Bioinformatics Centre, The Chinese University of Hong Kong, Shatin, New Territories, Hong Kong; Hong Kong Institute of Diabetes and Obesity, The Chinese University of Hong Kong, Shatin, New Territories, Hong Kong; Department of Biochemistry and Molecular Genetics, Feinberg School of Medicine, Northwestern University, Chicago, Illinois 60208, USA; Department of Computer Science and Engineering, The Chinese University of Hong Kong, Shatin, New Territories, Hong Kong; Hong Kong Bioinformatics Centre, The Chinese University of Hong Kong, Shatin, New Territories, Hong Kong; Hong Kong Institute of Diabetes and Obesity, The Chinese University of Hong Kong, Shatin, New Territories, Hong Kong; Sanford Burnham Prebys Medical Discovery Institute, La Jolla, California 92037, USA

**Keywords:** structural variation, cancer, optical DNA mapping

## Abstract

Structural variations (SVs) are commonly found in cancer genomes. They can cause gene amplification, deletion and fusion, among other functional consequences. With an average read length of hundreds of kilobases, nano-channel-based optical DNA mapping is powerful in detecting large SVs. However, existing SV calling methods are not tailored for cancer samples, which have special properties such as mixed cell types and sub-clones. Here we propose the Cancer Optical Mapping for detecting Structural Variations (COMSV) method that is specifically designed for cancer samples. It shows high sensitivity and specificity in benchmark comparisons. Applying to cancer cell lines and patient samples, COMSV identifies hundreds of novel SVs per sample.

## BACKGROUND

Structural variations (SVs), such as large insertions, deletions, inversions, translocations and copy number variations, are commonly found in cancer genomes [1–3]. Their high prevalence is due to a combination of factors, including defects in the DNA replication and repair pathways and inefficient apoptotic response [4–7]. SVs in cancer genomes can cause a variety of functional consequences, including altered protein sequence, gene loss, change of gene copy number, gene fusion, perturbed epigenetic signals and gene regulation and change of 3D genome structure [8–11]. Furthermore, some SVs are recurrent in particular cancer types [12–14], rendering them potentially useful for cancer diagnosis and classification.

It is challenging to detect large SVs by short read sequencing due to difficulties in read alignment and determination of the whole genomic span affected by an SV, especially when the break points are within tandem repeats or when the SV involves DNA contents not contained in the reference [15, 16]. As such, it has been shown advantageous to use different experimental technologies to detect SVs of different sizes and complexities [10]. Among the technologies currently available, optical DNA mapping [17, 18], due to its long read length (hundreds of kilobases on average), is powerful for detecting large SVs. The low cost also makes optical DNA mapping an attractive option as compared with long-read sequencing.

In nano-channel-based optical mapping systems [18], specific sites on the DNA are labeled either by single-stranded enzymatic nicking followed by repair with fluorescent dye conjugated nucleotides, or by direct labeling methods without nicking DNA [e.g. Bionano Genomics’s Direct Label and Stain (DLS) protocol]. The labeled DNA molecules are linearized and imaged in nanometer-scaled channels. The positions of the fluorescent labels on each molecule define a signature of the molecule, which can be compared with the expected label positions derived from a reference genome to determine the genomic location from which the molecule originated and identify any differences between the DNA molecule and the aligned region of the reference.

Based on this idea, a few methods have previously been proposed for calling SVs from optical mapping data [19–21]. These methods have been extensively applied to identify SVs from human samples [22–24]. However, all these methods were originally developed for non-cancer samples, and are thus not suitable for cancer samples due to their unique properties, as follows.

First, cancer samples usually contain a mixture of other cell types in addition to cancer cells, such as cells from adjacent non-cancer tissues, immune cells and stromal cells [25]. As a result, for an SV specific to cancer cells, among all the DNA molecules from that locus, only those coming from the cancer cells would contain information about the SV. This fraction of SV-supporting molecules can be small when the tumor content in the sample is low, and it is further halved for a heterozygous SV. An SV calling method developed for non-cancer samples can easily miss these SVs if it wrongly assumes that all the cells have the same genetic makeup and expects each SV to have around 100% or 50% of the aligned molecules supporting it in the cases of a homozygous and heterozygous SV, respectively. This issue is further aggravated by the presence of cancer sub-clones, each of which can contain a different set of SVs.

Second, it is common for cancer genomes to have abnormal copy numbers at different scales, from specific focal genomic regions (e.g. extrachromosomal circular DNAs) to whole chromosomes (aneuploidy). Therefore, the proportion of SV-supporting molecules can vary substantially along the genome. An SV calling method designed for non-cancer samples would fail to detect many SVs if it wrongly assumes that the proportion of SV-supporting molecules distributes around two constant mean values, $x\%$ and $x/2\%$, respectively, for all homozygous and heterozygous SVs in the whole genome, even if it allows $x$ to take a value other than 100.

Third, as compared with non-cancer genomes, cancer genomes usually contain substantially more SVs and the SVs are more complex. Accordingly, the resulting alignments and *de novo* assemblies of optical mapping data usually contain more errors, which can lead to inaccurate SV calls for SV identification methods that depend heavily on the reliability of the alignments or assemblies.

Realizing these challenging properties of cancer genomes, here we propose a new method, Cancer Optical Mapping for detecting Structural Variations (COMSV), which is specifically designed for cancer samples. We show that it has higher precision and sensitivity as compared with existing SV callers. We also demonstrate the use of COMSV in accurately calling SVs from both cancer cell lines and patient tumor samples.

In addition, we propose a new scheme for SV annotation that provides more information than the ones used by the previous SV calling methods, taking into account redundancy of reported SVs (e.g. not to report a duplication also as an insertion), uncertainty of SV break points (e.g. distinguishing between fully identified SVs and those having only partial break point information) and potential functional consequences of the SVs.

## METHODS

COMSV includes a pipeline for identifying insertions and deletions (the ‘indel pipeline’) and a pipeline for identifying other types of SVs (the ‘complex SV pipeline’). A high-level description of the pipelines is given below, whereas the details are provided in Supplementary Methods.

**Figure 1:**
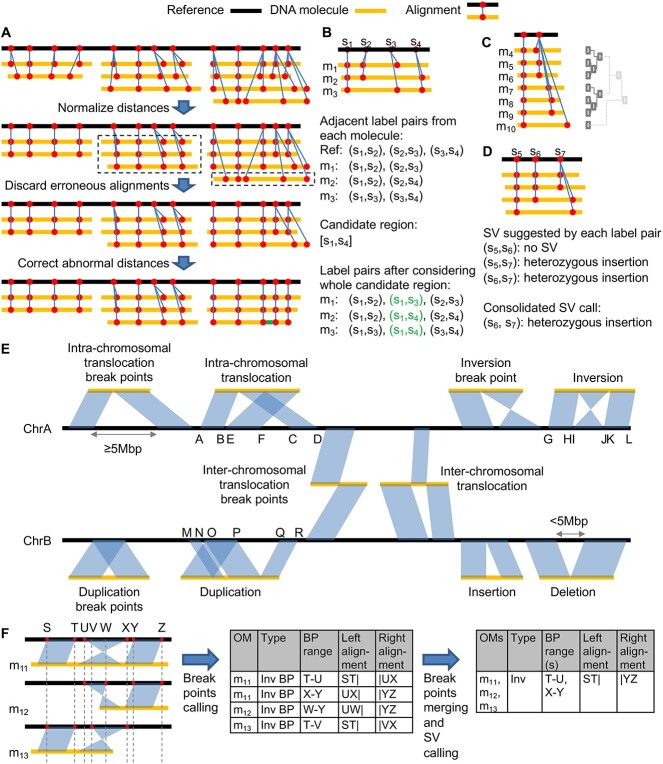
The COMSV pipelines, which include the indel pipeline (A–D) and the complex SV pipeline (E–F). **A**) Observed-to-expected distance ratios between two labels can deviate from 1 due to scaling or alignment errors (first row). After normalization, for alignments that still have these abnormal ratios (second row, dashed rectangles), the mutually reinforcing ones are considered indel candidates, while the others are discarded (third row). Isolated label pairs with abnormal distances are instead corrected (fourth row, green bar). **B**) Adjacent label pairs are collected from individual molecules to form candidate indel regions based on their overlaps, which enables the collection of distances between non-adjacent labels from a molecule if they are adjacent in another molecule (shown in green). **C**) For each label pair, the collected distances are clustered to identify the number of distinct alleles and the number of molecules that support each of them. **D**) For each potential indel region, the clustering result of each label pair suggests an initial SV type, and the final SV type of the whole region is determined by considering these suggestions jointly. **E**) Different types of SVs that can be identified from split-alignments. An SV break point is called if a split alignment suggests an SV event, while a complete SV is called if the full span of the SV can be determined. **F**) An example of calling SV break points and complete SVs. The reference is repeatedly displayed multiple times to show the split-alignment of each molecule clearly. The vertical dotted lines mark key aligned labels that define the boundary of each aligned segment on the reference. The corresponding labels on the reference are shown in red. Break point information is first collected from individual molecules and then considered together to refine the break point locations and determine whether complete SVs can be called.

The indel pipeline, which is based on alignment results, consists of four main steps (Figure 1A–D), namely (1) processing of the resulting alignments, (2) extraction of inter-label distances, (3) clustering of the inter-label distances and initial identification of indels and (4) post-processing of the indel calls. Each of these steps involves designs customized for cancer samples.

COMSV takes as input the alignment of optical mapping data of individual DNA molecules to a reference map produced by the *in silico* digestion of a reference genome. In the first step (Figure 1A), COMSV normalizes the distances between labels on the molecules to correct for incomplete molecular linearization [21]. After normalization, if the distance between two adjacent labels on a molecule deviates substantially from that on the reference, there could be an indel or the alignment could be wrong. Depending on whether multiple molecules display similar distance deviations consistently, COMSV either identifies the region as having a potential indel, considers the alignment as wrong and discards it or corrects the specific distance value according to the consensus. These three alternative treatments are crucial for cancer data, which have a relatively high error rate in the optical map alignments.

In the second step (Figure 1B), all pairs of adjacent labels on the reference genome and on the aligned molecules are extracted. Conceptually, by comparing the observed distances between two labels on the molecules and their expected distance on the reference, insertions and deletions can be identified. However, unlike previous methods that consider each label pair separately, COMSV groups the overlapping ones into candidate indel regions and considers all pairs of labels within each region at the same time. By doing so, molecules with the labels closest to SV break points missing can also be involved in calling an SV, which provides more information for accurate indel calling. Redundancy is also reduced by avoiding the same SV break point from being repeatedly called from different label pairs. In addition, this design reduces the negative effects of incorrect molecule alignments to the SV calling procedure.

In the third step (Figure 1C), for each label pair, their distances on the aligned molecules are clustered. In most situations, these distances form either one single cluster or two clusters, the former of which corresponds to having no indel or a homozygous indel, while the latter corresponds to having a heterozygous indel. However, due to mixed cell types, sub-clones and copy number changes, the proportion of molecules that support the indel allele is not known *a priori* and the flexibility of the clustering approach enables highly sensitive detection of indels regardless of the exact proportions. COMSV then determines both the SV type and zygosity of each potential indel according to the clustering results.

In the fourth step (Figure 1D), the initial indels identified in the third step are further processed to remove redundancy and determine the consensus span. Each resulting indel is then given a confidence score based on a model obtained by machine learning, which helps prioritize the identified indels for experimental follow-ups.

The complex SV pipeline uses split-aligned molecules, as in previous methods, partially unaligned molecules and contigs assembled *de novo* from individual molecules to identify SVs (Figure 1E-F). A split-aligned molecule is one with different parts of it aligned separately to different regions of the reference. Based on the locations and orientations of these different sub-alignments, the type of each candidate SV can be inferred (Figure 1E). Since it is generally difficult for alignment algorithms to make accurate split-alignments, COMSV also analyzes molecules that are partially aligned to the reference, and checks if the unaligned parts signal the possibility of an SV. For example, if the unaligned part can be aligned to the same reference location as the aligned part, there could be a tandem duplication. This re-alignment approach of COMSV can identify additional SVs missed by the split alignments. Finally, aligning contigs to the reference provides very long-range information required by some SV types such as translocations.

For all the SV candidates identified from the split-alignments and partial alignments, the information collected from different molecules is further integrated to produce a final set of non-redundant SVs with detailed annotations (Figure 1F).

The details of the pipeline are provided in Supplementary Methods. We also provide COMSV as an open-source tool, available freely at https://github.com/kevingroup/COMSV.

## RESULTS

### Comparisons between COMSV and other methods

We compared COMSV with three existing methods for identifying SVs from optical mapping data, namely Bionano Solve [19], OMIndel [20] and OMSV [21], first using simulated indels with the proportion of molecules from the SV locus that support the SV allele varying from 10 to 90% (Figure S1).

In terms of precision (i.e. the ratio of SVs called by a method that are true), all methods had stable performance when the proportion of SV-supporting molecules was reasonably large, but COMSV best maintained the high precision when the proportion of SV-supporting molecules was lower than 15%, no matter zygosity was ignored (Figure S1a) or considered (Figure S1b). This shows that the other methods were unable to distinguish between true SV cases and sizing/alignment errors when the proportion of SV supporting molecules is low.

In terms of recall (also called sensitivity, i.e. the ratio of true SVs that are called by a method), all methods performed better when the proportion of SV-supporting molecules was large, but the performance of COMSV deteriorated much more slowly than OMIndel and OMSV when the proportion decreased (Figure S1c). When correct zygosity was required, COMSV outperformed all the three other methods when the proportion of SV-supporting molecules was large (Figure S1d).

Overall, COMSV achieved the best balance between precision and recall, as indicated by its consistently high F1 score for all proportions of SV-supporting molecules (Figure S1e,f).

Next, we produced a set of simulated data with mixed cell types. Specifically, based on a cell evolution graph (Figure S2), we simulated SVs in four groups of cells, namely normal cells (Sample 1), trunk cancer cells (Sample 2) and cancer cells in two sub-clones (Samples 3 and 4). Due to the evolutionary relationships between these cells, Samples 2, 3 and 4 contain all the SVs of Sample 1, while Samples 3 and 4 contain most SVs of Sample 2. We then produced optical mapping data from each of these four pure cell types, as well as three cell mixture scenarios of increasing complexity (Table S1), namely a cancer sample with high tumor content (Sample 5), a cancer sample with low tumor content and one sub-clone (Sample 6) and a cancer sample with low tumor content and two sub-clones (Sample 7).

**Figure 2:**
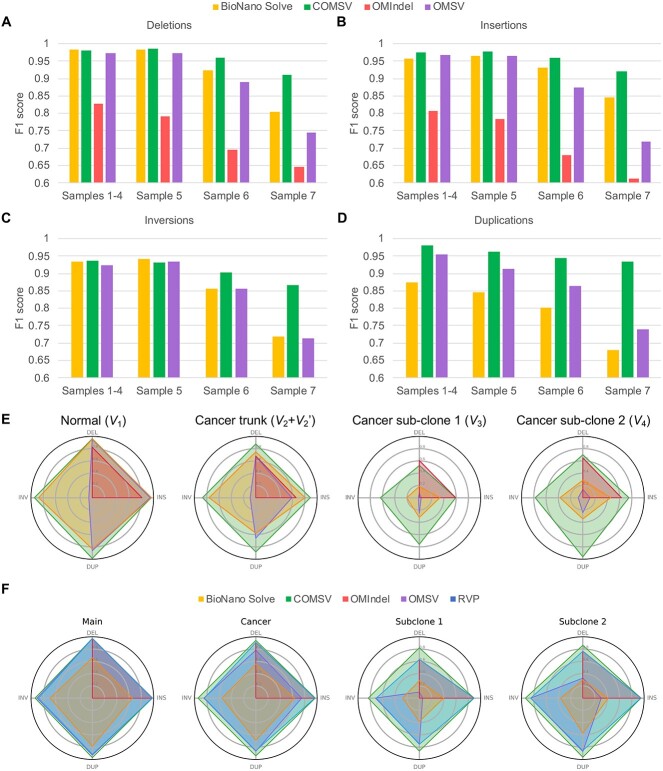
Comparisons between the performance of COMSV and three existing SV calling methods based on simulated data with different cell compositions based on a nicking enzyme (A–D) or DLS (E). **A–D**) For each type of SVs, including deletions (A), insertions (B), inversions (C) and duplications (D), the performance of each method when applied to the homogeneous cell samples with one cell type per sample (Samples 1–4) and the heterogeneous cell samples with multiple cell types per sample (Samples 5–7) is shown. Since OMIndel could only identify indels, it is omitted from Panels C and D. **E–F**) F1 scores of the different methods in detecting SVs in Sample 7 introduced at different stages during cancer evolution for Nt.BspQI (E) and DLS (F) labeling.

We evaluated the performance of the four methods based on each type of SVs they identified from each sample (Figure 2). For the homogeneous cell samples (Samples 1–4), the performance of COMSV was comparable with Bionano Solve and OMSV in calling deletions, insertions and inversions (Figure 2a-c), which is expected since Bionano Solve and OMSV were originally designed for normal samples that do not have mixed cell types. For duplications, COMSV outperformed Bionano Solve and OMSV (Figure 2D), partially attributable to its targeted re-alignment of unaligned parts of molecules.

The strengths of COMSV are more clearly demonstrated by its performance on the heterogenous cell samples (Samples 5–7), especially the most complex sample with two sub-clones (Sample 7). COMSV outperformed all the other methods substantially for all four types of SVs in this sample (Figure 2A–D). When the SVs introduced at different stages during the simulated cancer evolution were investigated separately, we found that the better performance of COMSV was largely due to SVs specific to the cancer cells, especially those in the sub-clones (Figure 2E). These results show that COMSV is more capable of handling the special properties of cancer samples, such as the presence of sub-clones, than the other methods.

To investigate how the SV calling performance of the different methods was affected by alignment accuracy, we supplied the correct molecule alignments as inputs to them (Figure S3). In general, all methods benefited from receiving these correct alignments. For example, OMSV became much more capable of identifying inversions and duplications when supplied with the correct alignments (comparing Figure 2C–D with Figure S3c–d). Yet importantly, the performance of COMSV was only minimally reduced when it was given the real, noisy alignments as compared with having the artificial, correct alignments, showing that it does not require alignments of very high quality as input. This is important since for real cancer samples, molecule alignments are expected to contain a high level of errors.

Besides, even when the correct alignments were supplied to all methods, COMSV still performed substantially better than the other methods when calling indels from the samples with cancer sub-clones (S6 and S7) (Figure S3a–b), suggesting that these other methods had additional limitations unrelated to their reliance on correct alignments.

Next, we compared COMSV with the Bionano rare variant pipeline (RVP), which was specifically designed for detecting SVs from optical mapping data with a low fraction of molecule support. Since this pipeline was mainly designed for optical mapping data produced from DLS, we produced a new set of simulated data based on the recognition sites of the Direct Labeling Enzyme 1 (DLE-1). As seen in Figure 2F, COMSV outperformed RVP and the other three methods on the DLS data, especially in detecting SVs specific to the cancer sub-clones. RVP performed better than the original Bionano Solve in detecting rare inversions and duplications specific to the cancer sub-clones, but it still failed to detect many insertions and deletions in sub-clone 2.

Overall, these results show that COMSV is able to detect SVs of various types with high precision and sensitivity, from optical mapping data labeled by either a nicking enzyme or DLS. The advantage of COMSV over the other existing methods is most prominent when detecting SVs supported by few molecules such as those contained only in specific cancer sub-clones.

### Identification of SVs from cancer samples

With the ability of COMSV in identifying SVs from synthetic data with cancer properties verified, next we applied it to identify SVs from real cancer samples. We obtained optical mapping data, including previously published data and some data newly generated in this study, from cancer and non-cancer cell lines and patient samples (Table S2). By comparing the SVs identified from the cancer and non-cancer samples, especially matched blood samples from the same patients, cancer-specific SVs could be identified. The matched non-cancer samples also helped evaluate the sensitivity of our SV calls, because most SVs identified from the non-cancer sample should also be detected in the corresponding cancer sample. Our pipelines support data produced by either nicking enzymes or DLS (Supplementary Methods).

**Figure 3:**
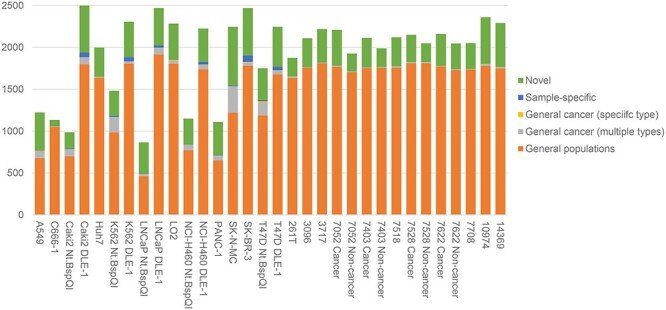
SVs identified by COMSV from the real samples. For each set of data, the identified SVs are put into different categories based on supporting evidence of them from independent data.

From each cancer sample, we detected 865–2525 SVs (Figure 3, Table S3). We verified the reliability of these SVs using two different methods. First, we checked whether the SVs have been previously reported in the general populations, in cancer samples, and in exactly the same samples but having the SVs detected using a non-optical mapping method (Figure S4, Supplementary Methods). From the results (Figure 3, Table S3), we found that 53.5–92.9% of the SVs were previously reported in non-cancer samples from general human populations. These SVs are therefore likely not cancer-specific but are simply alleles not contained in the reference genome. Among the remaining SVs, some were previously reported in cancer samples by the Pan-Cancer Analysis of Whole Genomes (PCAWG) consortium, including SVs identified only from the same cancer/tissue type of our samples and those that were also identified from other cancer/tissue types. These SVs constituted up to 14.1% of all SVs we found in a sample. For some of our samples, we also had SVs previously identified using other types of data, including short-read sequencing, linked-read sequencing and Hi-C (Table S7). Among the SVs we identified from each of these samples, there were 159–1029 of them reported in these previous studies (Table S8). Overall, on average $\sim $80% of the SVs we identified from each sample have direct support from at least one of these three types of previous studies. In contrast, when we took the SVs identified by COMSV from all the DLE-1 and Nt.BspQI data sets and moved them to random locations in the genome, only 22.1% and 35.9% overlapped with these previously reported SVs, respectively (Table S3), showing that the fraction of SVs identified by COMSV from the samples having independent support is much higher than this count- and size-matched random SV set. On the other hand, we have also identified a large number of novel SVs, at an average of 375 of them per sample. As a community resource, SVs identified from individual samples are provided in Supplementary File 1 and a unified non-redundant list of all the novel SVs is provided in Supplementary File 2. These novel SVs were likely missed by the previous studies because of their cancer-type specificity (some cancer types of our sample are missing in PCAWG, such as nasopharyngeal carcinoma), sample specificity and difficulties in identifying them without the long-range information provided by optical maps.

Next, we compared the SVs identified from matched cancer and non-cancer samples obtained from the same patients. For the four tongue squamous cell carcinoma samples, optical mapping data from blood samples of the same patients were also produced. Calling SVs from each sample independently and then comparing these SV call sets (Table S4), we found that the call sets of the tumor and blood samples from each patient are more similar to each other than to either cancer or blood call sets from other patients. This observation is consistent with the large proportion of non-cancer-specific SVs we found from the samples, as discussed above. Furthermore, in all four pairs of samples, we found that the proportion of SVs called from the blood samples that were also called from the matched cancer samples (86.8–90.0%) is larger than the proportion of SVs called from the cancer samples that were also from the matched blood samples (77.2–85.3%) (Table S4), which is in line with the expectation that the cancer samples contain somatic SVs not present in the matched blood samples.

**Figure 4:**
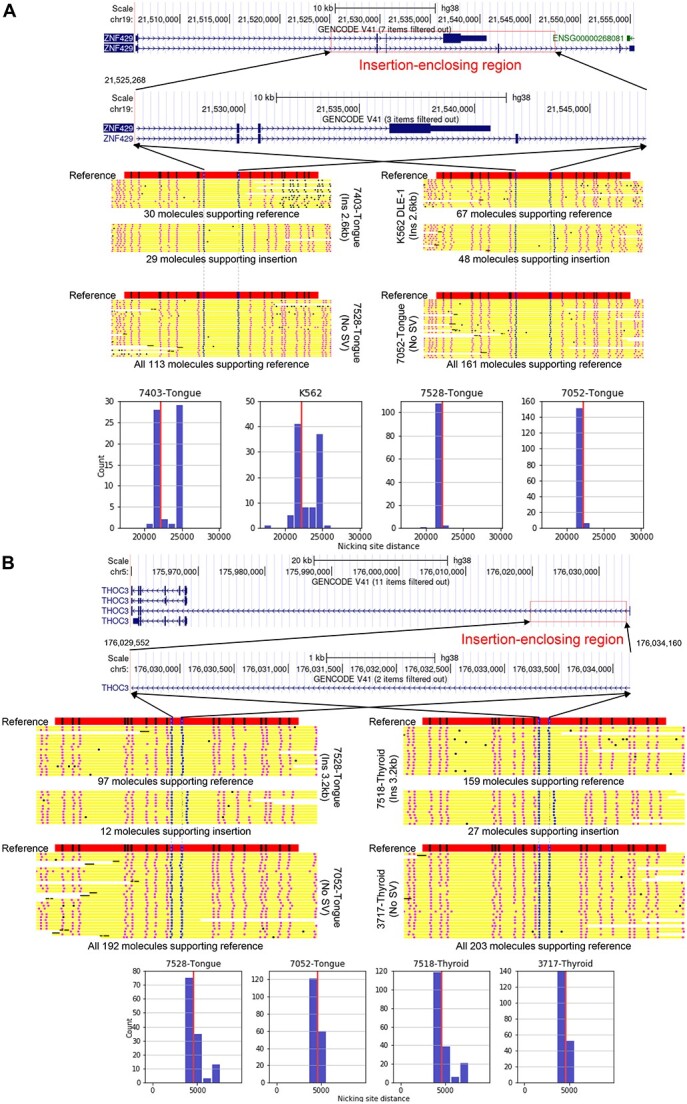
Examples of insertions called, from chromosome 19 (A) and chromosome 5 (B). In each panel, the top part shows the detected location of the insertion and a gene that it overlaps. The middle part shows the expected label pattern in the locus and the observed label patterns on optical maps from samples that have the insertion called or not called. The blue, pink and black dots on a molecule correspond to an anchor label, a non-anchor aligned label and an unaligned label, respectively. If the number of molecules aligning to the locus is too large, only the molecules with the largest and smallest distances between the anchoring labels are shown. The lower part shows the observed distance between the two anchoring labels between which the insertion is called. The expected distance according to the reference genome is marked with a red vertical line.

To illustrate the different types of SVs identified by COMSV from the cancer samples, we visualize the alignments involved in several examples. Figure 4 shows two examples of novel insertions called from the cancer samples not reported in the other sources that we analyzed. Figure 4A shows a 2.6kb insertion on chromosome 19. This insertion was called in five samples, including 7403 Cancer, 10974, Caki2, K562 and LNCaP. From the molecule alignments in 7403 Cancer and K562 (Figure 4A), it is clear that the insertion is heterozygous in both samples, although the fraction of molecules that support the insertion is closer to 50% in 7403 Cancer than in K562 (which is <40%). In contrast, in samples 7052 Cancer and 7528 Cancer from which the insertion was not called, all molecules aligning to this locus do not support the presence of an insertion. Histograms of the distance between the anchoring labels also clearly show a bimodal distribution of distances in 7403 Cancer and K562, but a unimodal distribution of distances close to the expected distance according to the reference genome in 7052 Cancer and 7528 Cancer. Based on the two anchoring labels between which the distance on the supporting molecules is larger than the expected distance on the reference, the insertion enclosing region (i.e. the region within which the insertion happens) overlaps the *ZNF429* gene. *ZNF429* was reported to have the highest mutation frequency (36% with missense mutations) in the rare thymoma and thymic carcinoma in a small study of 14 samples [26], but otherwise there were few former reports of mutations of this gene in cancer. Our finding that this SV is contained in five cancer types from different tissues (tongue squamous cell carcinoma, hepatocellular carcinoma, kidney clear cell carcinoma, chronic myelogenous leukemia and prostate adenocarcinoma) may indicate a previously unappreciated role of this novel SV and the *ZNF429* gene in cancer.

Similarly, Figure 4B shows a 3.2kb insertion on chromosome 5 that is identified in four samples, including 7518 Cancer, 7528 Cancer, LNCaP and SK-BR-3. Again, the insertion is found to be heterozygous in 7518 Cancer and 7528 Cancer, but only 27/186=15% and 12/109=11% of the aligned molecules support the insertion in these samples, respectively. Considering that (a) the absolute numbers of supporting molecules (27 and 12) are still fairly large, (b) the quality of these alignments is good, as judged by the consistency of their label patterns and the expected pattern on the reference genome and (c) the insertion was called independently in four samples, the insertion calls should be reliable. This example illustrates COMSV’s ability to identify SVs even when the fraction of SV-supporting molecules is very low.

**Figure 5:**
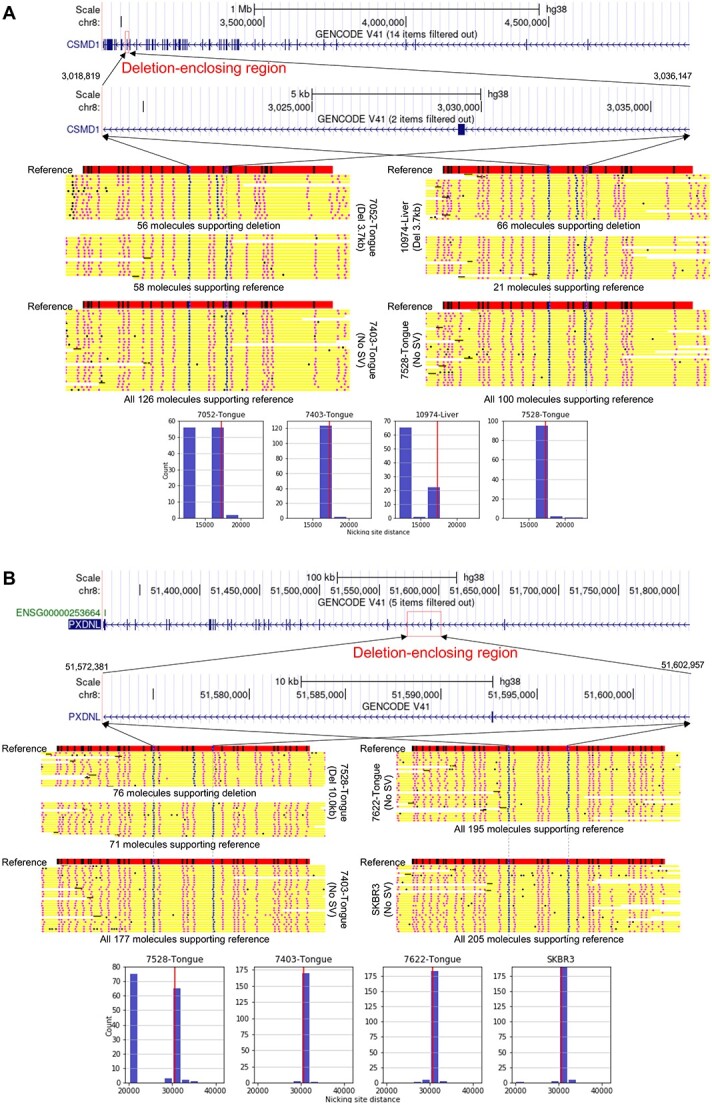
Examples of deletions called, both from chromosome 8. In each panel, the top part shows the detected location of the deletion and a gene that it overlaps. The middle part shows the expected label pattern in the locus and the observed label patterns on optical maps from samples that have the insertion called or not called. The blue, pink and black dots on a molecule correspond to an anchor label, a non-anchor aligned label and an unaligned label, respectively. If the number of molecules aligning to the locus is too large, only the molecules with the largest and smallest distances between the anchoring labels are shown. The lower part shows the observed distance between the two anchoring labels between which the deletion is called. The expected distance according to the reference genome is marked with a red vertical line.

Figure 5 shows two examples of novel deletions called from the cancer samples not reported in the other sources that we analyzed. Figure 5A shows a 3.7kb deletion on chromosome 8. The deletion was called from two samples, including 7052 Cancer (tongue squamous cell carcinoma) and 10974 (hepatocellular carcinoma). In both samples, there is a clear bimodal distribution of distances between the two anchoring labels, although the fraction of molecules supporting the deletion is lower in 7052 Cancer (56/114=49%) than 10974 (66/87=76%) (Figure 5A). The deletion-enclosing region overlaps the gene *CSMD1*, which is a tumor suppressor gene in breast cancer [27] and esophageal squamous cell carcinoma [28].

Figure 5B shows a large, 10.0kb deletion on chromosome 8 that was found only in sample 7528 Cancer. The molecule alignments clearly show that the deletion is heterozygous in this sample.

**Figure 6:**
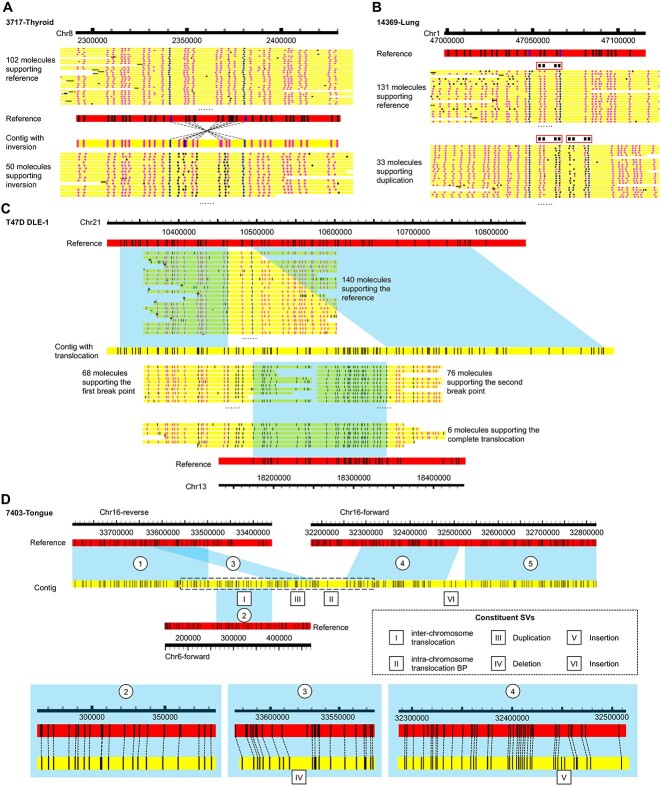
Examples of complex SVs called. **A**) An inversion called on chromosome 8. In addition to the individual molecules, the contig assembled from the molecules and its alignment with the reference are also shown. **B**) A tandem duplication called on chromosome 1. Each copy of the duplicating unit is highlighted by a red rectangle. **C**) An inter-chromosomal translocation called on chromosomes 21 and 13. Segments of a contig and individual molecules aligned to the reference are indicated by blue parallelograms. In all three panels, the blue, pink and black dots on a molecule correspond to an anchor label, a non-anchor aligned label and an unaligned label, respectively. If the number of molecules aligning to the locus is too large, only a sample of the molecules is shown. **D**) A very complex set of SVs called on chromosomes 16 and 6. Aligned segments of the contig are indicated by blue parallelograms and numbered (1–5). The individual constituent SVs are indicated by roman numerals (I–VI). The box with dashed line boundaries indicates the region with molecule alignments shown in Figure S5.

Finally, Figure 6 shows four examples of complex SVs called from the cancer samples. Figure 6A shows an inversion identified on chromosome 8 from the thyroid anaplastic carcinoma sample 3717. 102/152=67% of the aligned molecules support the reference allele, while the remaining 50/152=33% of the aligned molecules support an inversion. These inversion-supporting molecules align well to a contig assembled *de novo* from the optical maps, which clearly shows an inverted pattern at the locus. This inversion is also identified from the bladder cancer sample 3096.

Figure 6B shows an example of tandem duplication identified on chromosome 1 from the lung pleomorphic carcinoma sample 14369. 131/164=79.9% of the aligned molecules support only one copy of the repeating unit, defined by a pattern of four labels. The remaining 33/164=20.1% of the aligned molecules show a second tandem copy of the repeating unit right next to the first copy.

Figure 6C shows an example of inter-chromosomal translocation identified from the breast ductal carcinoma cell line T47D. A contig assembled *de novo* from the molecules has a left segment and a right segment aligned to adjacent loci on chromosome 21, while the middle segment between them is aligned to chromosome 13. There are 140 molecules whose alignments to the reference span the two loci without any split alignment, thus supporting the reference allele. On the other hand, there are 68 molecules that are split-aligned to the first segment on chromosome 21 and to chromosome 13, which support the first set of translocation break points. Similarly, there are 76 molecules split-aligned to the second segment on chromosome 21 and to chromosome 13, which support the second set of translocation break points. Finally, there are six molecules whose alignments cover the first segment on chromosome 21, followed by chromosome 13, followed by the second segment on chromosome 21, and therefore they support the whole translocation. Interestingly, this translocation (or some of its break points) is also identified from 15 other samples (Huh7, LNCaP DLE-1, LO2, H460 DLE-1, 261T, 3096, 3717, 7052 Cancer, 7403 Cancer, 7518, 7528 Cancer, 7622 Cancer, 7708, 10974 and 14369). The large number of samples from which the translocation signals are independently detected and the presence of molecules that support the whole translocation event make this a very reliable translocation call.

Finally, Figure 6D shows a very complex set of SVs identified from the tongue squamous cell carcinoma sample 7403 Cancer. The contig assembled *de novo* from the molecules is partly aligned to chromosome 16 and partly aligned to chromosome 6, which form an inter-chromosomal translocation (I). The two segments of the contig aligned to chromosome 16 are aligned to different regions of chromosome 16 in opposite orientations, thus forming an intra-chromosomal translocation (II). The first segment shows patterns of a duplication (III), with a duplicated copy containing a deletion (IV). The other segment shows patterns of two insertions (V and VI). Since the reliability of this complex set of SVs depends on the correctness of the contig, we checked the alignments of individual molecules to it and found that it is supported by a large number of aligned molecules (Figure S5).

Overall, these examples demonstrate the ability of COMSV in calling SVs of different types and zygosities, including those with only a small proportion of supporting molecules.

## DISCUSSION

Despite recent advances in long-read sequencing technologies and the release of new high-throughput platforms (such as the PacBio Revio system), optical DNA mapping still has the longest average read length of over 300kb from routine procedures. The cost of optical DNA mapping is also relatively low (US$500 for 500$\times $ coverage of the human genome) as compared with long-read sequencing, making it a preferred platform for detecting large SVs. Together with low-coverage sequencing data, the precise break points at base resolution could also be determined. For example, optical mapping can be first used to confidently identify rough locations of SVs, and then a local assembly of sequencing reads can be performed to improve the precision of break points detection, as previously demonstrated [21].

For the SVs identified by COMSV from each of the human samples, we found supports from previous studies for an average of 80.0% of them (ranging from 57.0–93.3%). These SVs were previously identified from the general population, cancer samples (specific to the same cancer/tissue type or also other cancer/tissue types) and the same samples (based on non-optical mapping methods). One limitation of this analysis is the uneven coverage of the samples in previous studies. Whereas some cancer types were extensively studied, some others (e.g. nasopharyngeal carcinoma) were missing from large-scale cancer studies such as PCAWG. In addition, some particular samples had SVs called using multiple types of experimental methods previously, while other samples had none. Despite these differences, in each sample at least 6.7% of the SVs identified by COMSV were not found in the previous studies considered. We believe this is due to a combination of both the use of optical DNA mapping, which provides long read length, and the higher sensitivity of COMSV as compared with other existing SV calling methods. It would be useful to further validate these SVs and investigate their functional significance, especially the ones that COMSV independently identified from multiple samples. As more optical DNA mapping data are produced from cancer samples, we also hope to apply COMSV to identify SVs from them to create a comprehensive catalog of large and complex SVs present in cancer genomes. COMSV can also be applied to other species with a high-quality reference genome, such as mouse, which is commonly used in cancer studies.

For the samples with both Nt.BspQI and DLE-1 data, the two sets of SVs overlapped but each also contained unique SVs. This could be due to a number of reasons. First, DLE-1 in general has a higher density of labels than Nt.BspQI, allowing it to provide more information for calling SVs in general. However, there are also some loci at which Nt.BspQI produces more labels than DLE-1 (Figure S6). Second, DLE-1 prevents double-strand breakage at ‘fragile sites’ caused by nearby nicks on opposite strands, leading to longer DNA molecules and thus better preservation of long-range information for SV calling. Third, due to different labeling densities and patterns, alignment and assembly methods optimized for one labeling method may not work well, or not without careful re-calibration, for the other labeling method. Alignment/assembly errors thus caused may have led to some of the inconsistencies in the two sets of SVs. Finally, although the optical mapping data sets involved in this study had high depth of coverage in general, some particular loci have lower coverage in the data for one labeling method than the other, which affects the ability to detect SVs at these loci, especially for the SVs with low allele ratio caused by sub-clones or cell type mixture.

## CONCLUSIONS

In this study, we have proposed the COMSV method for identifying SVs based on optical DNA mapping data produced from cancer samples, which is difficult due to cell type mixture, presence of sub-clones, aneuploidy and other copy number changes, large number of SVs and complex SVs. We have shown that COMSV achieves good precision and sensitivity even if the SV allele is only observed in a small proportion of molecules aligned to the locus. Applying COMSV to cancer cell lines and patient samples, we have identified various novel SVs not reported in previous studies, including SVs that we recurrently but independently called from multiple samples. We have provided the SVs identified from the human samples, which cover a wide range of tissue and cancer types, as a community resource for studying cancer genomes.

**Key Points**

- Identifying structural variations from cancer samples is difficult due to cell type admixture, sub-clones, aneuploidy, and large number of complex variations.
- We propose COMSV, which is specifically designed for identifying structural variations from optical DNA mapping data produced from cancer samples.
- COMSV uses a flexible algorithm that can handle the challenging properties of cancer samples.
- We show that COMSV has high precision and recall in detecting various types of structural variations.
- Using COMSV, we identified structural variations from a compendium of cancer cell lines and patient samples from various cancer types.

## Supplementary Material

COMSV_new_revised_suppl_bbad520

SupplementaryFile1_bbad520

SupplementaryFile2_bbad520

SupplementaryFile3_bbad520

## Data Availability

The datasets used and/or analyzed during this study are available from the corresponding author on reasonable request.

## Notes

**Le Li** was a PhD student in Department of Computer Science and Engineering at the Chinese University of Hong Kong.

**Chenyang Hong** was a PhD student in the Department of Computer Science and Engineering at the Chinese University of Hong Kong.

**Jie Xu** was a PhD student in Department of Biochemistry and Molecular Genetics at Northwestern University.

**Claire Yik-Lok Chung** was a PhD student in School of Life Sciences at the Chinese University of Hong Kong.

**Alden King-Yung Leung** was a PhD student in School of Life Sciences at the Chinese University of Hong Kong.

**Delbert Almerick T. Boncan** was a PhD student in School of Life Sciences at the Chinese University of Hong Kong.

**Lixin Cheng** is an associate professor at Shenzhen People's Hospital.

**Kwok-Wai Lo** is a professor in the Department of Anatomical and Cellular Pathology at the Chinese University of Hong Kong.

**Paul B. S. Lai** is a professor in the Department of Surgery at the Chinese University of Hong Kong.

**John Wong** is a consultant in the Department of Surgery at the Chinese University of Hong Kong.

**Jingying Zhou** is an assistant professor in School of Biomedical Sciences at the Chinese University of Hong Kong.

**Alfred Sze-Lok Cheng** is a professor in School of Biomedical Sciences at the Chinese University of Hong Kong.

**Ting-Fung Chan** is an associate professor in School of Life Sciences at the Chinese University of Hong Kong.

**Feng Yue** is a professor in Feinberg School of Medicine at Northwestern University.

**Kevin Y. Yip** is a professor in NCI-Designated Cancer Center at Sanford Burnham Prebys Medical Discovery Institute.
