# Supplementary material for: Accurate identification of structural variations from cancer samples
Source: Brief Bioinform. 2024 Jan 16;25(1):bbad520. doi: 10.1093/bib/bbad520 (PMC10794023; doi:10.1093/bib/bbad520)
Supplement: COMSV_new_revised_suppl_bbad520 [file comsv_new_revised_suppl_bbad520.pdf]

S1 Supplementary Methods

S1.1 Details of the indel pipeline

Before running COMSV pipeline to call SVs, optical mapping data of individual DNA molecules are aligned to a reference. For all the results in this study obtained from nicking-enzyme-based optical mapping data, molecules were aligned using OMBlast [1] (in OMTools [2] v1.4a). For the results obtained from DLS, molecules were aligned using both OMBlast and RefAligner [3] (in Bionano Solve v3.5.1), with additional details given below. In both cases, in the alignment result, each molecule is either (fully or partially) aligned to a single genomic location, split-aligned with different parts aligned to different locations, or unaligned. For each aligned or split-aligned molecule, each label is either aligned to exactly one label on the reference or unaligned.

For each aligned molecule, COMSV first corrects for potential scaling errors as follows (Figure 1a). For every two adjacent labels on the molecule, the distance between them on the molecule divided by the distance between the two corresponding aligned labels on the reference is referred to as the observed-to-expected distance ratio. Each of these ratios is then divided by the median of all the observed-to-expected distance ratios from this alignment. For a perfectly linearized DNA molecule with all the labels correctly aligned, all these ratios should be close to one, with the small deviations caused by measurement errors alone. For a DNA molecule with imperfect linearization but a constant scaling factor along the whole molecule, after this normalization, all the ratios should also be close to one. Therefore, in both cases, if after the normalization there is a high proportion ($> a\%$) of distance ratios strongly deviated from one ($< 1 - \gamma$ or $> 1 + \gamma$), where a and γ are user parameters, the alignment is deemed low confident and marked for potential rejection (the whole list of parameters of COMSV and their default values are given in Table S5).

Whereas some of these low-confidence alignments can be incorrect alignments, some others can have abnormal distance ratios because of the presence of SVs instead. To distinguish between these two cases, the aligned genomic locations of these low-confidence molecules are analyzed. Specifically, all low-confidence alignments with overlapping aligned locations are grouped. For groups with more than N alignments, they are treated as reinforcing each other, considering the fact that the alignment of each molecule was performed independently. For such reinforced cases, all the alignments in the group will be "rescued", while the other low-confidence alignments will be discarded.

The second step (Figure 1b) aims at collecting observed-to-expected distance ratios for the identification of indels in the later steps. Since indels can have different sizes and can occur at different locations, it would be ideal to check the distance between all pairs of labels no matter they are adjacent or not. However, computationally this is not feasible since it would require checking many label pairs. Instead, COMSV first starts with all pairs of labels that are adjacent either on the reference or on at least one molecule. For each of these label pairs, among all molecules containing both labels (according to their alignments to the reference), if at least p_d of them have an observed-to-expected distance ratio strongly deviating from one (larger than 1+δ or smaller than 1-δ, where δ is the minimum SV ratio) and the absolute differences are larger than s_{min} (minimum SV size), a suspected indel case is called. All these suspected indel regions are collected and the overlapping ones are grouped. Finally, for each group, all the label pairs within this region, no matter adjacent or not, will be considered in

the calculation of distance ratios.

In the third step (Figure 1c), for each pair of labels collected in the second step, their distances on the molecules are clustered using complete-link hierarchical clustering until no two distance values respectively from any two clusters have an absolute difference smaller than s_{min} or a ratio smaller than δ. Complete-link clustering is used because it is most sensitive to large-value outliers. The resulting clusters are tight, and outliers can be easily identified. When this clustering is finished, clusters that contain less than n_s member molecules are considered outliers and are removed. The remaining clusters go through one more round of clustering using median-link hierarchical clustering (i.e., distance between two clusters defined as difference between their medians), which considers the opinions of all members in a cluster more evenly, to determine the number of clusters. Each final cluster is expected to contain molecules that contain a specific allele of the genomic region involved.

In the fourth step (Figure 1d), the clustering result for each pair of labels is first used to determine the SV type. Specifically, based on the number of clusters n_c and the average observed-to-expected distance ratio s_i of each cluster i, the following rules are used to determine the SV type:

Number of clusters	Average distance ratios	Conclusion
$n_c = 1$	$s_1 \gg 1$	Homozygous insertion
$n_c = 1$	$s_1 \approx 1$	No SV
$n_c = 1$	$s_1 \ll 1$	Homozygous deletion
$n_c = 2$	$s_1 \gg 1$ and $s_2 \gg 1$	Multi-allelic double insertion
$n_c = 2$	$s_1 \gg 1$ and $s_2 \approx 1$	Heterozygous insertion
$n_c = 2$	$s_1 \gg 1$ and $s_2 \ll 1$	Multi-allelic insertion-deletion
$n_c = 2$	$s_1 \approx 1$ and $s_2 \ll 1$	Heterozygous deletion
$n_c = 2$	$s_1 \ll 1$ and $s_2 \ll 1$	Multi-allelic double deletion
$n_c > 2$		Complex SV

In these rules, a distance is considered significantly smaller than ($\ll$) or larger than ($\gg$) a threshold if the normalized deviation is larger than s_{min} in the respective direction.

All these initial indel-spanning regions are then grouped to identify redundant cases. Specifically, all the indel-spanning regions are sorted according to their starting locations. All regions that have a reciprocal overlap of >50% are linked, and finally all regions directly or indirectly linked are grouped together. From each group, one final indel is produced with its type and zygosity determined by a simple majority vote of all the initial indels, and its span determined by following the indel candidate with the highest coverage (number of aligned molecules covering the region) among all the candidates in the group. Final indels with a coverage less than c are considered low-confidence and are discarded.

S1.2 Confidence scores of detected indels

We then trained a support vector machine model with a radial basis function (RBF) kernel for distinguishing the correct indel calls of COMSV from the incorrect ones. Specifically, for each indel identified by COMSV from the simulation data, we put it into one of three possible classes by comparing it with the ground truth, namely wrong call (the indel does not actually exist), correct call of indel location but wrong zygosity, and correct call of indel location and zygosity. For each indel, three features were derived based on the outputs of COMSV, namely the ratio of molecules that support the SV allele, the detected indel size, and

the number of molecules aligned to the locus. All features were transformed to have a value range between 0 and 1, where this was done by subtracting the minimum followed by dividing by the maximum. Based on 10 runs of class-stratified five-fold cross-validations, the trained model achieved an average area under the receiver-operator characteristic (AUROC) of 0.98 (stdev=0.01) in distinguishing between true and false indels (without considering zygosity). In contrast, models that involved single features alone achieved an average AUROC of 0.84 (stdev=0.03), 0.94 (stdev=0.02), and 0.78 (stdev=0.03), with the ratio of molecules that support the SV allele, SV size, and the number of molecules aligned to the locus, respectively. Since the model has a prediction score for each of the three classes, these scores can also be used to check whether it is more likely for an SV to be homozygous or heterozygous.

S1.3 Details of the complex SV pipeline

The complex SV pipeline of COMSV uses split-alignments of molecules and partially unaligned molecules to identify SVs. In a split-alignment, two or more segments of a molecule are aligned to the reference non-consecutively. Depending on where the different segments are aligned and the orientations of these individual alignments, different types of SVs are called (Figure 1e). In general, if a molecule can provide information about one break point of an SV but not the complete information of the whole SV, only an SV break point is called.

The exact way that SV break points and complete SVs are called depends on the SV type (Figure 1e). For a translocation, there should be three break points in total for a complete event, namely two break points between which a DNA segment is cut, and a third break point at which the segment is inserted. If a molecule contains two segments split-aligned to two regions on the same chromosome separated by at least 5Mb, two intra-chromosomal translocation break points (marked as "-BP" in the final SV lists) are called. Specifically, suppose a segment is aligned to region $[A, B]$ and a later segment is aligned to region $[C, D]$ in the same alignment orientation, with $A < B < C < D$, then two break points are called between B and C, with one expected to be close to B and the other one expected to be close to C. The actual translocation can involve either a segment that starts near C and ends at an unknown location after D translocated to a location near B, or a segment that starts at an unknown location before A and ends near B translocated to a location near C. Now, suppose after the second segment, there is a third segment on the molecule aligned to the region $[E, F]$, with $B < E < F < C$, then a complete translocation event is reported with a segment that starts near C and ends near D translocated to a location between B and E. A similar logic can be used to call a complete intra-chromosomal translocation if the third segment is before the first segment rather than after the second segment. Inter-chromosomal translocations can also be called in a similar way, except that the translocated segment should come from a chromosome different from the one at which it is inserted.

For an inversion, there should be two break points in total for a complete event, between which the DNA is inverted. Suppose a segment is aligned to region $[G, H]$ and a later segment is aligned to region $[I, J]$ in the reverse alignment orientation, with $G < H < I < J$, then one inversion break point is called and its location is between H and I. The inversion should involve a segment that starts at this break point and ends at a location after J. Now, suppose after the second segment, there is a third segment on the molecule aligned to the region $[K, L]$, with $K > J$, then a complete inversion is called with the inverted segment starting at a location between H and I and ending at a location between J and K. In some

cases, the inverted segment has a symmetric pattern that makes it difficult for the aligner to detect an inversion using the split-alignment strategy. If there are clear distance changes of the labels flanking the inverted segment, it is possible for COMSV to detect an insertion and a deletion instead (Figure S7). To detect these cases, we collected the deletion-insertion pairs with genomic distance less than 20kb and size difference less than 5%. These pairs are re-considered as inversions if they concurrently appear in at least 2 molecules.

For a duplication, there should be three break points in total for a complete event, namely two break points between which a DNA segment is duplicated, and a third break point at which the segment is inserted. If a molecule contains two segments with their aligned regions on the reference overlapping each other, the starting and ending locations of their overlapped region are called as two duplication break points, where the segment duplicated should completely enclose this region. Other parts of the molecule can help refine these break points and identify the third break point. For example, suppose the overlapped region of the two alignments is $[O, P]$, if the segment on the molecule between these two aligned segments is aligned to region $[M, N]$ on the reference, where $M < N < O < P$, then the duplicated region may start somewhere between N and O. On the other hand, if a later segment of the molecule is aligned to region $[Q, R]$ on the reference, where Q is much larger than P, then the duplicated DNA is expected to have inserted to a location near Q.

A special case of duplications is that the whole molecule is aligned to consecutive regions on the reference except that two adjacent segments are aligned to the same region. In this case, a tandem duplication is called.

Finally, if different segments of a molecule are aligned to nearby genomic regions within 5Mb, an insertion or deletion will be called depending on the aligned locations of the segments. Some of these indels may have already been identified by the indel pipeline, but some may only be identified by this complex SV pipeline especially in the case of a large deletion, where the different segments cannot be aligned to the reference without a split-alignment.

The complex SV pipeline of COMSV also makes use of partially aligned molecules to identify duplications and inversions. Specifically, for each molecule that is only partially aligned to the reference, the unaligned segment is extracted and aligned to the reference again by itself. It may be uniquely aligned to a single region on the reference or to multiple different regions. In the former case, if the new alignment has an orientation different from the original aligned segment, an inversion break point or a complete inversion would be called, and if the new alignment region overlaps the one of the original aligned segment, a duplication would be called. On the other hand, if the alignment is not unique to a single region and one of these aligned regions supports neither an inversion nor a duplication, no SVs would be called.

It should be noted that in the above descriptions, break points can only be identified up to the closest aligned label, and therefore calling a break point precisely requires both the presence of a label (e.g., a nicking site) near the break point and a correct alignment of the label on at least one molecule.

If there are multiple complex SV break points that support overlapping SVs of the same type, they are grouped together to give one final break point call, with the possible range of the break point defined in three different ways, all reported in the output file. The three ways are: 1) taking the intersection of all the individual break point ranges, 2) taking the minimum span that covers all the individual break point ranges, and 3) taking the break point range with the highest molecule support. For example, for the inversion shown in

Figure 1f, the molecules m_{11} and m_{13} propose the left break point to be within the regions $T-U$ and $T-V$, respectively, and therefore in the final call, the common region of them, $T-U$, is taken as the range of the left break point.

For translocations and duplications, if the translocated/duplicated content is inverted when inserting to the new location, the SV type is recorded as "invert-translocation" and "invert-duplication", respectively.

It is possible that some duplications are initially missed or identified as insertions if the duplicated parts are not aligned in the initial alignment. To deal with these cases, COMSV includes a module for "rescuing" unaligned segments of optical maps that have other parts properly aligned. Specifically, for each unaligned segment of an optical map, we check whether its label pattern matches any region on the reference near (<500kb) the aligned parts. If ≥80% of the unaligned segment can match well, a duplication would be called. Similarly, if the reverse of the label pattern can match the reference of a nearby region well, an inversion would be called. All SVs identified by re-alignment of initially unaligned segments are marked as "-realigned" on the output SV list.

It is possible that some very complex types of SV appear in cancer genomes, such as nested SVs with one SV completely enclosing another SV. These cases can be identified for manual inspection and analysis by looking for regions with a high density of SVs identified by COMSV.

S1.4 Customized methods for DLE-1 data

Compared to traditional optical mapping data using a nicking enzyme (such as Nt.BspQI) for labeling, DLS data using the DLE-1 enzyme for labeling have conceptually no fragile sites (i.e., sites easily having double-stranded breakage due to two nicks on opposite strands close to each other). As a result, in DLS data the molecules are longer on average. There is also a higher density of labels. These properties created difficulties for the alignment algorithms we used (especially OMBlast), which were originally developed for nicking enzyme-based data.

To deal with this issue, COMSV integrates SVs detected from multiple sets of alignments when working on DLS data, as follows.

Indel detection requires highly accurate alignment of individual labels. OMBlast was not optimized for DLS data and could produce alignments with high error rates. In contrast, RefAligner and Assembler in the Bionano Solve toolset have been optimized for DLS. Therefore, for DLS data, we used two sets of alignments produced by RefAligner in our indel pipeline, namely i) direct alignment of individual molecules to the reference, and ii) alignment of assembled contigs to the reference. Since the assembled contigs already represent a form of consensus among the individual molecules, when calling indels based on their alignments, we did not impose the requirements for molecule alignments (N, n_s, and c). For an identified SV, if there was at least one contig that supported the reference allele, we called it a heterozygous SV. Otherwise, we called it a homozygous SV.

To identify complex SVs from DLS data, in addition to the two types of alignments used to detect indels, we also engaged OMBlast to do a two-round alignment of individual molecules, which is a strategy we also used in our previous studies [4, 5]. In the first round, molecules were aligned to the reference with the option of complex SV break points detection disabled, such that molecules that required only simple alignments could be performed reliably, while some molecules that required more complex alignments (such as those around SV break

point regions) were either completely unaligned or had segments of them unaligned. In the second round, all unaligned molecules and molecule segments were aligned to the reference again, independent of the alignment results in the first round. The final complex SV callset was produced by applying our complex SV pipeline to all alignments (these two rounds of OMBlast alignment, RefAligner direct molecule alignment, and RefAligner contig alignment). These three sets of SVs were then integrated by including 1) all complex SVs detected from the RefAligner contig alignments, 2) all duplications detected from RefAligner direct molecule alignments (because RefAligner in Bionano Solve could not generate split alignments and thus could not be used to detect SVs such as inversions), and 3) SVs detected from OMBlast two-round split alignments of individual molecules, except for those that required the rescue procedure for unaligned segments as described above.

To improve the accuracy of our callset, we removed indels that overlapped any duplications or inversions detected by the complex SV pipeline, because the confidence of local alignments around complex SV breakpoints was generally lower and the indel pipeline was more likely to generate false positive calls.

S1.5 Defining the overlap between an SV and another SV or a genomic region

In various parts of this study, we had to compare the location information of an SV with that of a simulated SV, an SV reported in a previous study, or an annotated gene.

To do that, we first needed to define the "spanning region" (or simply "span") of an SV, which is basically where the SV appears. Conceptually, the span of an insertion is the single nucleotide position at which the extra DNA sequence is inserted, the span of a deletion is the genomic region deleted, the span of an inversion is the genomic region inverted, and the span of a duplication is the genomic region duplicated. In a translocation, a genomic region is translocated to another region, which involves a deletion and an insertion, and thus it has two spans. Among the two spans of a translocation, the one with a smaller starting point (earlier chromosome, based on the order of 1, 2, ..., 22, X, Y, or same chromosome but smaller coordinate) is called the first span and the other one is called the second span.

In all these cases, due to the nature of optical mapping, a span of an SV can only be estimated based on the innermost pair of labels that enclose it. The region between these two labels on the reference was the practical definition of a span used by COMSV.

Accordingly, we defined different types of overlaps that involve an SV as follows:

- For non-translocations, two SVs of the same type are considered overlapping if their spans have reciprocal overlaps of $\geq$50%.
- For non-translocations, an SV is considered overlapping with a genomic region (e.g., a gene or a gold-standard SV, which has a precise span rather than an estimated one) if the span of the SV has an overlap with the genomic region of $\geq$1bp.
- Two translocations are considered overlapping if the shortest distance between their first span is $\leq$500kb and the shortest distance between their second span is $\leq$500kb.
- A translocation is considered overlapping with a genomic region if the shortest distance between the genomic region and either span of the translocation is $\leq$50kb.

In comparisons involving SV locations, we sometimes also considered additional requirements, such as the size of an SV. For example, even if two insertions have the same span (i.e., estimated insertion point), they would be different insertions if the lengths of their inserted sequences are very different. For parts of our study that introduced such additional requirements, the details are provided in the corresponding method descriptions separately.

S1.6 Procedures for generating simulated data

In the first simulation experiment, we generated large indels with the proportion of supporting molecules ranging from 10% to 90% as follows. First, following the parameter settings of a previous work [5], we simulated a list of indels on the hg38 human reference genome using pIRS [6]. We then took the reference genome as one haplotype and the indel-added genome as another haplotype to simulate molecules using *in silico* digestion to 100x average genome coverage from each haplotype, using the same parameter settings as in a previous work [5]. Finally, molecules from these two sets were randomly sampled at different rates to produce 17 data sets, respectively corresponding to sampling 10%-90% of the molecules from the reference haplotype and the remaining from the indel-added haplotype, with an increment of 5% each time.

In the second simulation experiment, we first generated the genomes of normal cells, trunk cancer cells, and cells in the two sub-clones by simulating different sets of SVs using pIRS based on the evolution graph (Figure S2). The actual numbers of SVs generated are shown in Table S6. We then simulated molecules for each genome to 100x average coverage. These molecules were then sampled to form the molecules of the seven samples according to their target cell compositions (Table S1).

The above data sets were simulated based on the Bionano Irys platform (which uses the Nt.BspQI enzyme for labeling). To check the performance of COMSV on data produced by the Saphyr platform (which uses the DLE-1 enzyme for labeling), we also generated simulated optical mapping data by applying DLE-1 *in silico* digestion to molecules of Sample 7 in Table S1.

S1.7 Application of the different SV callers on the simulated data

We compared COMSV with four other SV callers on the simulated data, namely the two SV callers included in Bionano Solve v3.5.1 (regular pipeline and RVP), OMIndel, and OMSV. For the regular Bionano Solve pipeline, the exact list of command-line arguments used was "$BIONANOSOLVE_DIR/Pipeline/1.0/pipelineCL.py -l prop${sample} -t $BIONANOSOLVE_DIR/RefAligner/1.0 -b $BNXPATH/${sample} -y -d -U -i 5 -F 1 -W 1 -c 1 -a optArguments_haplotype_saphyr_human.xml -r hg38_BSPQI_700.cmap -f 0.2 -J 48 -j 60 -jp 80 -T 80 -N 6". We ran the RVP pipeline via the Bionano Access platform with the same set of arguments used in the regular pipeline. For OMIndel, we tried different values of the minimum supporting reads and picked the one that gave the highest F1 score across samples, which was found to be 7. Default values were used for all other parameters. When zygosity was considered, the minimum cluster size of the reference reads was set to 10, as suggested by the author. For OMSV, we set the minimum support to 10 and the minimum likelihood ratio to $1e4$, which provided the best performance on the simulated data.

After obtaining the callsets of the different SV callers, we compared them with the gold-standard list of SVs after removing SVs that overlapped fragile sites, regions with no sequence information on the reference, and pseudo-autosomal regions. A called SV was considered correct if it overlapped with a gold-standard SV of the same type with the estimated SV size within 5 times of the correct size. For complex SVs, we also required each called break point to be within 20kb from a break point of the gold-standard SV. Precision, recall, and F1 score were then computed accordingly.

S1.8 Collection of published optical mapping data

We collected published optical mapping data from several sources (Table S2). All published cell line data with DLE-1 labeling were from a previous study [7] and kindly provided to us by Vineet Bafna in the form of BNX files. The C666-1 data were produced in our previous work [5] and we used the alignment files from that study directly. All other published cell line data with Nt.BspQI labeling were produced in our previous work [8] and we took the BNX files as our input. The published patient sample data were from a previous study [9] and kindly provided to us by James Broach, with the help of Lijun Zhang, in the form of BNX files.

S1.9 Production of optical mapping data from cell lines and primary samples

The two immortalized human liver cell lines, Huh7 and LO2, were maintained in Dulbecco's modified Eagle's medium (Gibco) with 10% FBS (HyClone). All cells were cultured at 37°C in a humidified chamber containing 5% CO2.

A HCC patient who underwent hepatectomy at the Prince of Wales Hospital (Hong Kong) was included in this study. The specimen was processed immediately after surgery and snap-frozen in liquid nitrogen for DNA extraction. The patient gave written consent on the use of specimen for research purposes. The study protocol was approved by the Joint Chinese University of Hong Kong-New Territories East Cluster Clinical Research Ethics Committee.

High molecular weight (HMW) genomic DNA was extracted from Huh7 and LO2 cells using Bionano Prep™ Blood and Cell Culture DNA (Part no. 80004; Bionano Genomics, USA) with the Cell Culture Protocol (Document no. 30026 Rev F), and from 261T using Bionano Prep Animal Tissue DNA Isolation Kit (Part no. 80002) (Bionano Genomics, USA) with the Soft Tissue Protocol (Document no. 30077 Rev C). In detail, cell suspension was obtained by resuspending cell line pellets or homogenized solid tumor sample in 1X phosphate-buffered saline (PBS) buffer solution. Cell suspension was then embedded in low-melting agarose gel plugs and treated with protease K and RNase A. The gel piece was then molten with heat and agarose. HMW DNA was recovered from the molten gel via drop dialysis, and fluorescently labelled to generate sequence motif-specific patterns following the Bionano DLS protocol (Document no. 30206 Rev F). Optical mapping data were generated on the Bionano Saphyr system (Part no. 60325) using Saphyr Chip G1.2 (Part no. 20319). In brief, fluorescently labeled HMW DNA molecules were automatically stretched and imaged within nanochannel arrays. Distances between labels were calculated from the images into raw molecule maps and *de novo* assembled into consensus maps by Bionano Access v1.4.2 and Bionano Tools v1.4.1.

S1.10 A non-redundant list of SVs identified from the real samples

For each real sample, COMSV produced a final list of SVs identified from it with various types of annotations, such as SV type, zygosity, ranges of genomic locations of the break points, and proportion of aligned molecules supporting the SV allele. Following our previous work [4], we excluded several classes of genomic regions from SV calling that make molecule alignment erroneous or even impossible (Supplementary File 3), including 1) regions with low density of Nt.BspQI or DLE-1 labels, defined as regions $\geq$100kb with $\leq$5 labels, 2) regions with unresolved sequences in the reference ("N" regions) $\geq$50kb, 3) centromeres, and 4) pseudo-autosomal regions. We also excluded the Y chromosome.

To annotate the genes that overlap each SV, we downloaded the latest release (release 43) of comprehensive human gene annotation from GENCODE [10] in GTF format (`https://ftp.ebi.ac.uk/pub/databases/gencode/Gencode_human/release_43/gencode.v43.annotation.gtf.gz`). We considered only annotations on chromosomes 1-22, X, and Y. We annotated each SV by all the genes that it overlapped, including both coding genes and non-coding genes. For each gene, we listed the Ensembl gene ID, gene name, and gene type. For SVs that spanned $\geq$5Mb, which may overlap many genes, we did not include their gene annotations.

Besides, we also integrated the SV lists from individual samples to produce an overall list of non-redundant SVs identified from all samples. To do that, we considered one SV type at a time. We collected all SVs of that type identified from all samples. For an SV type other than translocations, overlapping SVs (as defined above) were put into the same group. This was done in a transitive manner, such that if the first and second SVs were grouped, the second and third SVs were grouped, and so on, until the k-th and $(k+1)$-th SVs were grouped, all $(k+1)$ SVs would all be grouped together. Each group was then represented by the SV in it that had the smallest genomic span. Due to this process of merging SVs across samples, in the final list of non-redundant SVs, we omitted annotations that required sample-specific information, such as zygosity and proportion of aligned molecules supporting the SV allele, but instead recorded whether a sample contained an original SV belonging to each group. We also simplified SV type annotations since the more specific types could be present in only some samples. For example, an "invert-duplication" (a duplication with the duplicated part inverted) was simply recorded as the more general "duplication" type.

Translocations were merged in a way slightly different from the other SV types due to their complexities. Conceptually, each translocation involves "cutting" a genomic region out and "pasting" it somewhere else. In practice, optical mapping data usually can only provide information about break points, by having different segments of a molecule aligned to different separated regions on the reference, without complete translocation information. Therefore, usually two different regions are identified to be involved in a translocation, but which one corresponds to the "cut" site and which to the "paste" site is not known. Because of this, when merging translocations called from different samples, both regions need to be considered. Two translocations were grouped only if both their first spans overlapped and their second spans overlapped. Procedure-wise, we sorted translocations based on their two spans' genomic locations, followed by grouping translocations with both spans overlapping using the transitive rule described above. Finally, for each set of merged translocation break points, it was considered not called from a sample if there are $\leq$4 molecules supporting it in the sample.

SV break points detected for which the complete SV could not be determined were marked as "-BP" in the output files.

S1.11 Identifying SVs called by COMSV from the real samples that were reported in previous studies

We collected multiple sets of SVs from different sources to check whether the SVs called by COMSV from the real samples were also reported in some previous studies.

Two SV sets were from general populations. The first set included SVs larger than 2kb in size identified from 156 samples across 26 human populations using optical DNA mapping [4]. The second set was the NCBI Curated Common Structural Variants in dbVar [11], available at `https://www.ncbi.nlm.nih.gov/dbvar/studies/nstd186/`. These two sets together covered SVs of different sizes identified across various human populations.

SVs larger than 1kb in size previously identified from cancer samples were collected from the latest open Pan-Cancer Analysis of Whole Genomes (PCAWG) consensus callsets for structural variants [12], available at `https://dcc.icgc.org/releases/PCAWG/consensus_sv`. This database contained SVs from 2,588 cancer samples, each annotated with a cancer type. For samples with multiple SV files, we merged them into a single SV list for each sample. We omitted 190 samples that were not annotated with a cancer type.

Finally, for some specific samples, we collected previously reported SVs (from the supplementary materials of corresponding publications) larger than 1kb in size that were identified using non-optical mapping methods (Table S7). Again, for each sample with multiple lists of SVs, we merged them into a single list.

Based on these SV sets, we classified each SV identified by COMSV into one of five possible classes (Figure S4). If an SV was reported in either of the two general population SV sets (by having overlapping locations, the same type, and size ratio within [1/5, 5] with any known SV in the population sets for indels, or having the same type with any known SV as well as all breakpoints located within 10kb of that known SV in the population sets for complex SVs), we classified the SV as being found in the general population. Otherwise, we checked if the SV was reported by PCAWG. If it was, then we further checked whether it was reported in at least one cancer type other than the cancer type of our sample, in which case the SV was classified as being found in multiple cancer types, or else it was classified as being found in a specific cancer type. If the SV was not reported by PCAWG, then we further checked the SVs previously reported from this same sample, if available, to classify the SV as sample-specific (and previously reported) or novel.

S2 Supplementary materials

S2.1 Supplementary tables

Sample	1	2	3	4	5	6	7
Normal cells	100×	0×	0×	0×	20×	20×	40×
Trunk cancer cells	0×	100×	0×	0×	80×	50×	20×
Cancer sub-clone 1 cells	0×	0×	100×	0×	0×	30×	20×
Cancer sub-clone 2 cells	0×	0×	0×	100×	0×	0×	20×

Table S1: Cell type composition of the different samples in the second simulation experiment. For each sample, the amount of optical maps produced from each cell type in terms of average genome coverage is shown.

Sample	Type	Organ/tissue	State	Labeling	Data source	Number of molecules aligned	Average length of aligned molecules (bp)
A549	Cell line	Lung	Cancer (adenocarcinoma)	Nt.BspQI	[8]	654,790	233,733
C666-1	Cell line	Nasopharynx	Cancer (nasopharyngeal carcinoma)	Nt.BspQI	[5]	843,123	244,074
Caki2	Cell line	Kidney	Cancer (clear cell carcinoma)	Nt.BspQI	[8]	570,985	253,025
Caki2	Cell line	Kidney	Cancer (clear cell carcinoma)	DLE-1	[7]	2,424,944	297,000
Huh7	Cell line	Liver	Cancer (hepatocellular carcinoma)	DLE-1	This study	1,629,774	269,006
K562	Cell line	Blood	Cancer (chronic myelogenous leukemia)	Nt.BspQI	[8]	654,569	260,717
K562	Cell line	Blood	Cancer (chronic myelogenous leukemia)	DLE-1	[7]	2,035,576	297,915
LNCaP	Cell line	Prostate	Cancer (adenocarcinoma)	Nt.BspQI	[8]	586,712	245,513
LNCaP	Cell line	Prostate	Cancer (adenocarcinoma)	DLE-1	[7]	1,550,011	298,291
LO2	Cell line	Liver	Non-cancer	DLE-1	This study	1,802,745	240,815
NCI-H460	Cell line	Lung	Cancer (non-small cell carcinoma)	Nt.BspQI	[8]	693,391	234,121
NCI-H460	Cell line	Lung	Cancer (non-small cell carcinoma)	DLE-1	[7]	1,755,220	305,155
PANC-1	Cell line	Pancreas	Cancer (pancreatic carcinoma)	Nt.BspQI	[8]	502,557	246,440
SK-N-MC	Cell line	Adrenal glands	Cancer (neuroblastoma)	Nt.BspQI	[8]	1,097,849	250,155
SK-BR-3	Cell line	Breast	Cancer (adenocarcinoma)	DLE-1	[7]	2,356,988	310,497
T47D	Cell line	Breast	Cancer (ductal carcinoma)	Nt.BspQI	[8]	738,468	298,548
T47D	Cell line	Breast	Cancer (ductal carcinoma)	DLE-1	[7]	2,048,956	295,377
261T	Patient sample	Liver	Cancer (hepatocellular carcinoma)	DLE-1	This study	1,545,556	216,254
3096	Patient sample	Bladder	Cancer	DLE-1	[9]	1,950,385	354,979
3717	Patient sample	Thyroid	Cancer (anaplastic carcinoma)	DLE-1	[9]	2,023,467	363,756
7052	Patient sample	Tongue	Cancer (squamous cell carcinoma)	DLE-1	[9]	2,051,159	346,126
7052	Patient sample	Blood	Non-cancer	DLE-1	[9]	1,897,051	313,543
7403	Patient sample	Tongue	Cancer (squamous cell carcinoma)	DLE-1	[9]	1,966,708	334,900
7403	Patient sample	Blood	Non-cancer	DLE-1	[9]	1,181,812	348,413
7518	Patient sample	Thyroid	Cancer (anaplastic carcinoma)	DLE-1	[9]	2,147,760	314,234
7528	Patient sample	Tongue	Cancer (squamous cell carcinoma)	DLE-1	[9]	1,434,288	368,872
7528	Patient sample	Blood	Non-cancer	DLE-1	[9]	2,150,859	320,253
7622	Patient sample	Tongue	Cancer (squamous cell carcinoma)	DLE-1	[9]	1,660,765	390,182
7622	Patient sample	Blood	Non-cancer	DLE-1	[9]	2,290,781	262,175
7708	Patient sample	Thyroid	Cancer (anaplastic carcinoma)	DLE-1	[9]	1,422,898	393,706
10974	Patient sample	Liver	Cancer (hepatocellular carcinoma)	DLE-1	[9]	2,171,181	337,681
14369	Patient sample	Lung	Cancer (pleomorphic carcinoma)	DLE-1	[9]	2,095,861	353,533

Table S2: Optical mapping data of human samples included in this study.

Sample	General populations		General cancer (multiple types)		General cancer (specific type)		Sample-specific		Novel		Total
A549	677	(55.36%)	86	(7.03%)	5	(0.41%)	3	(0.25%)	452	(36.96%)	1,223
C666-1	1,053	(92.86%)	4	(0.35%)	0	(0%)	1	(0.09%)	76	(6.70%)	1,134
Caki2 Nt.BspQI	699	(70.89%)	88	(8.92%)	0	(0%)	7	(0.71%)	192	(19.47%)	986
Caki2 DLE-1	1,797	(71.17%)	86	(3.41%)	1	(0.04%)	53	(2.10%)	588	(23.29%)	2,525
Huh7	1,635	(81.83%)	12	(0.60%)	1	(0.05%)	NA		350	(17.52%)	1,998
K562 Nt.BspQI	984	(66.40%)	186	(12.55%)	0	(0%)	14	(0.94%)	298	(20.11%)	1,482
K562 DLE-1	1,805	(78.24%)	28	(1.21%)	0	(0%)	50	(2.17%)	424	(18.38%)	2,307
LNCaP Nt.BspQI	463	(53.53%)	27	(3.12%)	0	(0%)	3	(0.35%)	372	(43.01%)	865
LNCaP DLE-1	1,916	(77.57%)	77	(3.12%)	3	(0.12%)	29	(1.17%)	445	(18.02%)	2,470
LO2	1,803	(78.94%)	42	(1.84%)	4	(0.18%)	NA		435	(19.05%)	2,284
NCI-H460 Nt.BspQI	771	(67.10%)	65	(5.66%)	4	(0.35%)	5	(0.44%)	304	(26.46%)	1,149
NCI-H460 DLE-1	1,738	(78.08%)	54	(2.43%)	3	(0.13%)	31	(1.39%)	400	(17.97%)	2,226
PANC-1	651	(58.65%)	57	(5.14%)	3	(0.27%)	4	(0.36%)	395	(35.59%)	1,110
SK-N-MC	1,220	(54.32%)	316	(14.07%)	0	(0%)	8	(0.36%)	702	(31.26%)	2,246
SK-BR-3	1,781	(72.11%)	40	(1.62%)	7	(0.28%)	75	(3.04%)	567	(22.96%)	2,470
T47D Nt.BspQI	1,188	(67.85%)	153	(8.74%)	16	(0.91%)	15	(0.86%)	379	(21.64%)	1,751
T47D DLE-1	1,675	(74.58%)	44	(1.96%)	8	(0.36%)	43	(1.91%)	476	(21.19%)	2,246
261T	1,636	(87.21%)	15	(0.80%)	2	(0.11%)	NA		223	(11.89%)	1,876
3096	1,758	(83.40%)	5	(0.24%)	0	(0%)	NA		345	(16.37%)	2,108
3717	1,809	(81.52%)	9	(0.41%)	0	(0%)	NA		401	(18.07%)	2,219
7052 Cancer	1,768	(80.04%)	15	(0.68%)	0	(0%)	NA		426	(19.28%)	2,209
7052 Non-cancer	1,704	(88.43%)	10	(0.52%)	0	(0%)	NA		213	(11.05%)	1,927
7403 Cancer	1,752	(82.88%)	9	(0.43%)	0	(0%)	NA		353	(16.70%)	2,114
7403 Non-cancer	1,757	(88.38%)	10	(0.50%)	0	(0%)	NA		221	(11.12%)	1,988
7518	1,759	(82.93%)	15	(0.71%)	0	(0%)	NA		347	(16.36%)	2,121
7528 Cancer	1,812	(84.28%)	13	(0.60%)	0	(0%)	NA		325	(15.12%)	2,150
7528 Non-cancer	1,810	(88.34%)	16	(0.78%)	0	(0%)	NA		223	(10.88%)	2,049
7622 Cancer	1,773	(82.01%)	7	(0.32%)	0	(0%)	NA		382	(17.67%)	2,162
7622 Non-cancer	1,729	(84.42%)	11	(0.54%)	0	(0%)	NA		308	(15.04%)	2,048
7708	1,736	(84.64%)	6	(0.29%)	0	(0%)	NA		309	(15.07%)	2,051
10974	1,780	(75.39%)	22	(0.93%)	1	(0.04%)	NA		558	(23.63%)	2,361
14369	1,748	(76.30%)	16	(0.70%)	1	(0.04%)	NA		526	(22.96%)	2,291
Shuffle DLE-1	6,172	(13.71%)	3,640	(8.08%)	NA		NA		35,213	(78.21%)	45,025
Shuffle Nt.BspQI	2,131	(22.17%)	1,349	(14.03%)	NA		NA		6,134	(63.80%)	9,614

Table S3: Numbers of SVs called from the human samples that overlapped with other SV call sets. For non-cancer samples, cancer-type-specific comparisons were performed based on the organ/tissue type. For samples without SV call sets previously obtained from non-OM data, their sample-specific overlap numbers are marked as “NA”. Translocations were excluded from the random sets because they cover two separate genomic spans instead of one.

	7052 Cancer	7052 Non-cancer	7403 Cancer	7403 Non-cancer	7528 Cancer	7528 Non-cancer	7622 Cancer	7622 Non-cancer
7052 Cancer	-	84.01%	63.03%	61.13%	62.66%	60.16%	61.41%	57.02%
7052 Non-cancer	86.76%	-	64.25%	63.47%	64.25%	62.45%	63.28%	59.17%
7403 Cancer	63.17%	62.75%	-	85.29%	62.89%	60.80%	62.98%	58.99%
7403 Non-cancer	64.84%	64.89%	89.25%	-	64.99%	64.01%	64.79%	62.50%
7528 Cancer	62.82%	62.91%	62.96%	61.64%	-	83.46%	62.82%	59.80%
7528 Non-cancer	66.19%	66.80%	65.94%	66.24%	90.03%	-	66.24%	65.49%
7622 Cancer	59.45%	59.41%	60.58%	58.91%	60.09%	58.27%	-	77.23%
7622 Non-cancer	63.12%	63.80%	65.52%	65.36%	65.67%	66.40%	88.59%	-

Table S4: Overlap of the SVs identified from the tongue squamous cell carcinoma samples and matched blood samples from the same patients. Each entry is the number of SVs commonly called from the two samples corresponding to the row and the column of the entry divided by the sample corresponding to the row, i.e. $x_{ij} = |S_i \cap S_j|/|S_i|$, where x_{ij} is the entry in the i-th row and the j-th column of the table, S_i and S_j are the SV lists of the i-th and j-th samples, respectively, and $|S|$ denotes the number of SVs in the set S. The largest value in each row is underlined.

Parameter	Description	Default value
a	Maximum percentage of observed-to-expected distance ratios strongly deviated from one for high-confidence alignments	75
γ	Parameter for defining an observed-to-expected distance ratio to be strongly deviated from one	$\max(\delta,\ s_{min}/d_{ref})$
N	Minimum number of alignments to form a reinforcement group	5
p_d	Minimum percentage of alignments with distance ratios strongly deviated from one for calling a suspected indel case	10
δ	Minimum ratio of distance change to support an indel	0.05
s_{min}	Minimum size of an indel	2,000bp
n_s	Minimum size for a cluster to be retained	3
c	Minimum number of aligned optical maps covering an indel region	10

Table S5: List of parameters of COMSV and their default values. The order of the parameters follows their descriptions in the Materials and Methods section. d_{ref} denotes the distance of the reference allele.

Genome (Size range)	Missing / Extra sites	Small indels (1bp-100bp)	Large indels (5kb-50kb)	Inversions (50kb-500kb)	Duplications (50kb-500kb)	pCNVs (150kb-1.5Mb)	Translocations (150kb-1.5Mb)
Normal	5,840	295,405	1,196	81	82	0	0
Trunk cancer	11,588	472,703	2,307	238	213	24	36
Sub-clone 1	17,170	615,213	2,636	270	269	36	74
Sub-clone 2	17,151	614,277	2,601	269	262	27	82

Table S6: Numbers of small and large variations introduced to each genome of the second simulation experiment. Missing/Extra sites are damaged/novel labels caused by single nucleotide variations. pCNV: copy number variations due to large dissociative molecule fragments that cause polyploidy in cells.

Cell line	Short Reads	10x linked-reads	Hi-C	Other data
A549	[8]	NA	[8]	NA
C666-1	[5]	NA	NA	NA
Caki2	[8]	NA	[8]	[8]
K562	[8]	NA	[8]	[8]
LNCaP	[8]	NA	[8]	NA
NCI-H460	[8]	NA	[8]	NA
PANC-1	[8]	NA	[8]	NA
SK-N-MC	[8]	NA	[8]	NA
SK-BR-3	[13]	[13]	NA	[13]
T47D	[8]	NA	[8]	[8]

Table S7: Sources of previously reported SVs identified from samples involved in this study using non-optical mapping data. Other data include PCR amplifications and the detection of SV-induced fusion genes based on RNA-seq. NA: not available

Data set	Sample	Number of SVs identified by COMSV that were reportedly using other technologies
A549	A549	159
C666-1	C666-1	240
Caki2 DLE-1	Caki2	544
Caki2 Nt.BspQI	Caki2	199
K562 DLE-1	K562	480
K562 Nt.BspQI	K562	255
LNCaP DLE-1	LNCaP	539
LNCaP Nt.BspQI	LNCaP	175
NCI-H460 DLE-1	NCI-H460	495
NCI-H460 Nt.BspQI	NCI-H460	233
PANC-1	PANC-1	195
SK-N-MC	SK-N-MC	1,029
SK-BR-3	SK-BR-3	302
T47D DLE-1	T47D	442
T47D Nt.BspQI	T47D	318

Table S8: Number of intra-chromosomal SVs identified by COMSV that were also reported previously by using other technologies as described in Table S7.

S2.2 Supplementary figures

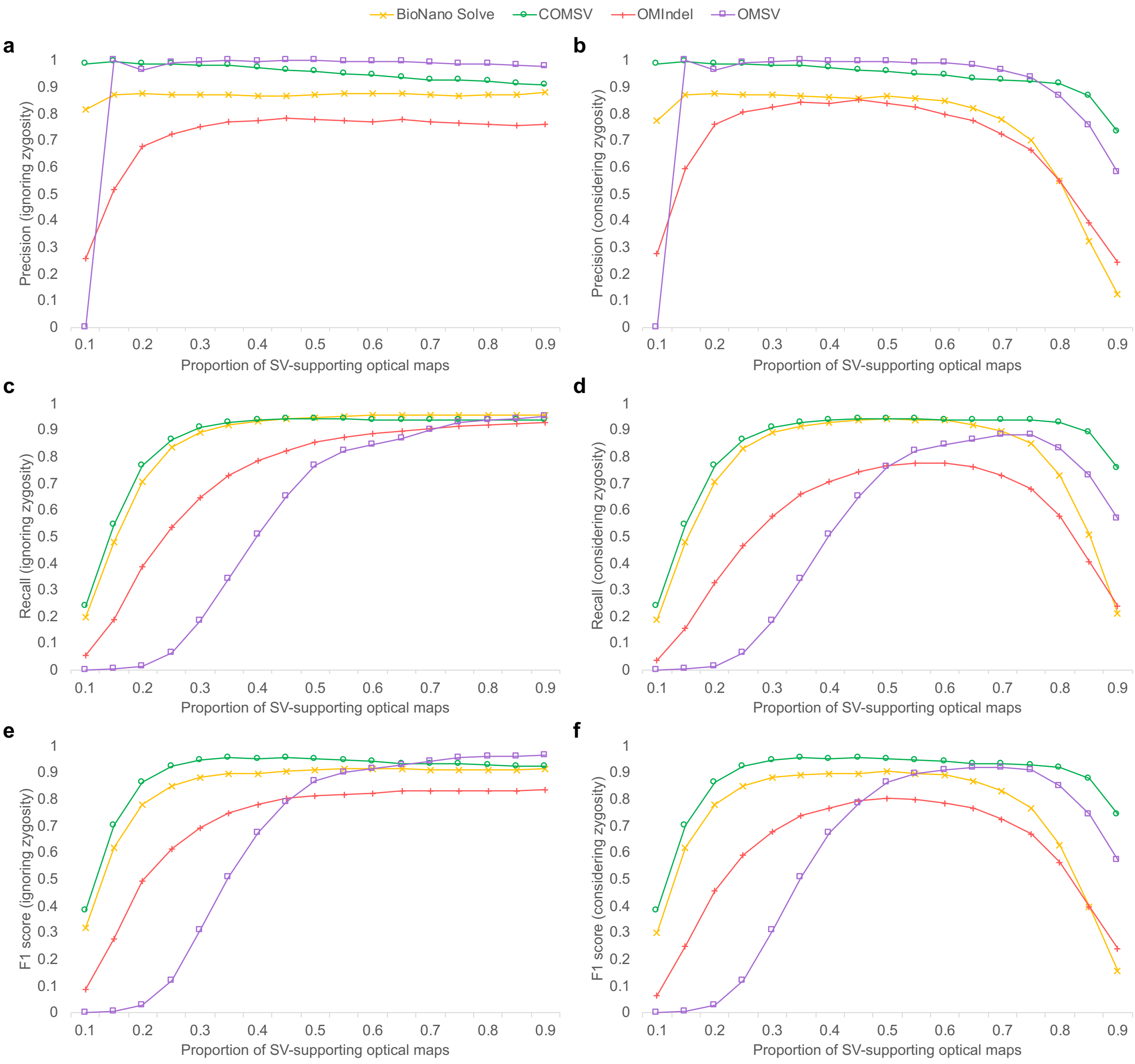

Figure S1: Comparisons between the performance of COMSV and three existing SV calling methods based on simulated data with a varying proportion of supporting optical maps at the SV loci. Precision (**a, b**), recall (**c, d**) and F1 score (**e, f**) of the different methods when the zygosity of the SVs are ignored (a, c, e) or considered (b, d, f) by the performance measures.

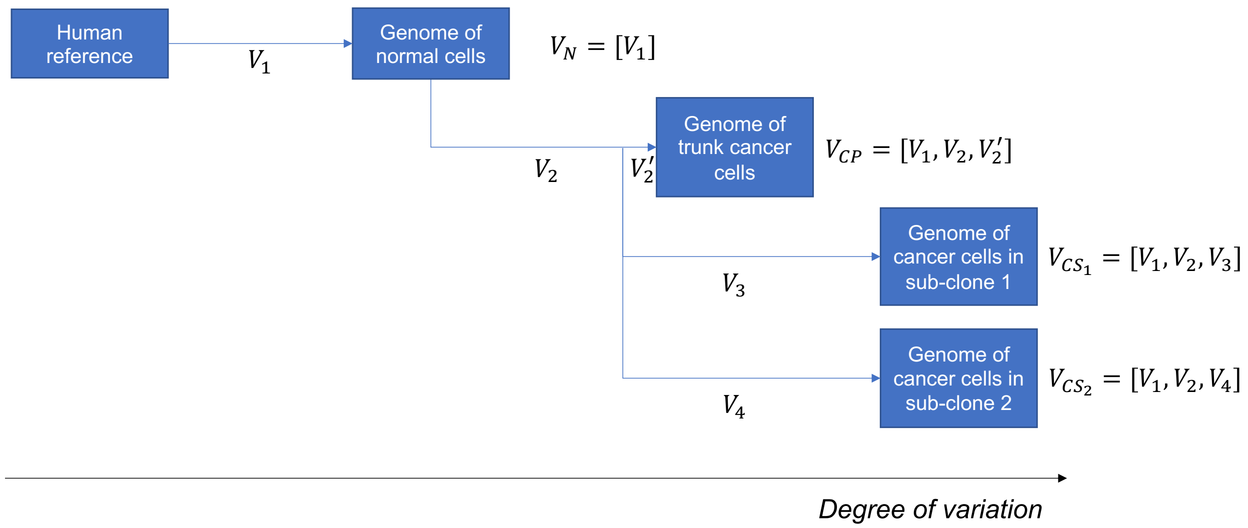

Figure S2: The evolution of cells in the second simulation experiment. Based on the human reference, a first set of SVs (V_1) were generated to create the genome of the normal cells. A second set of SVs (V_2) were then generated to create the genome of trunk cancer cells. While the trunk cancer cells continued to evolve with a small additional set of SVs (V_2'), two cancer sub-clones were evolved with their own sets of SVs (V_3 and V_4, respectively). The final sets of SVs contained in the genomes of the normal cells, trunk cancer cells, cells in the first sub-clone and cells in the second sub-clone are denoted as V_N, V_{CP}, V_{CS_1} and V_{CS_2}, respectively.

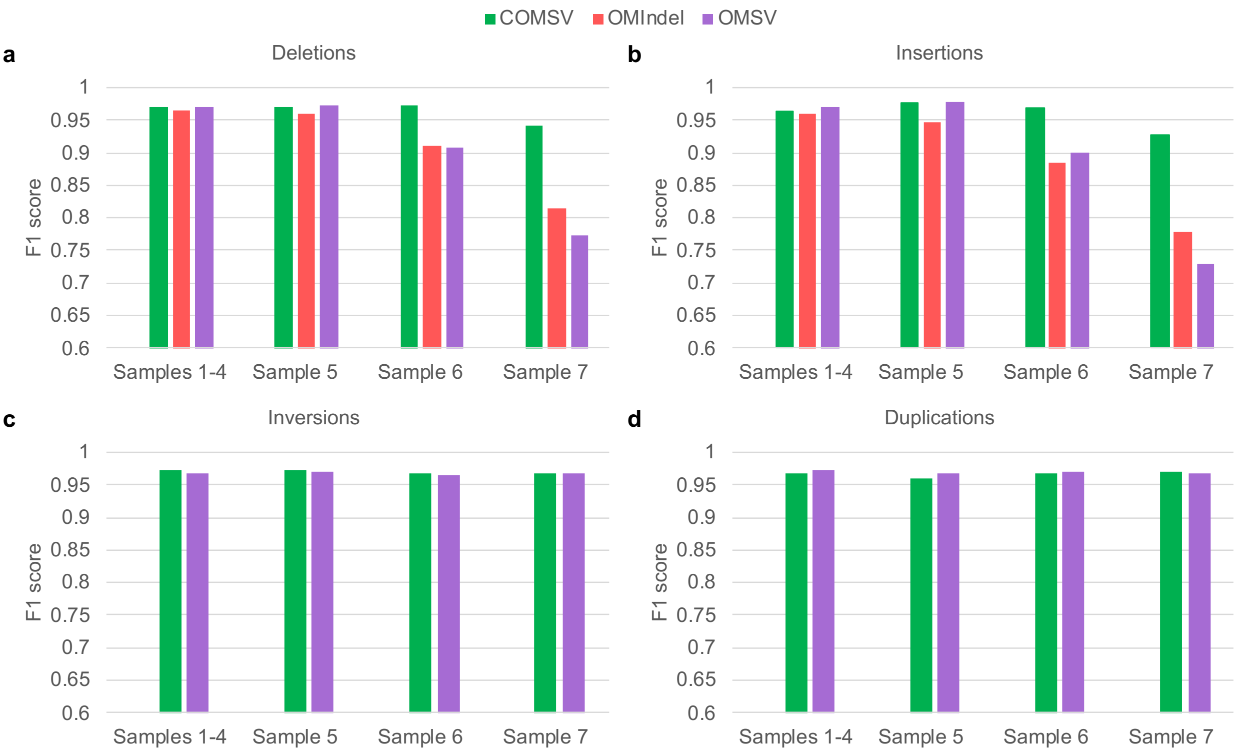

Figure S3: Comparisons between the performance of COMSV and other existing SV calling methods based on simulated data with different cell compositions, with the correct optical map alignments supplied as inputs. **a-d** For each type of SVs, including deletions (a), insertions (b), inversions (c) and duplications (d), the performance of each method when applied to the homogeneous cell samples with one cell type per sample (Samples 1-4) and the heterogeneous cell samples with multiple cell types per sample (Samples 5-7) is shown. Since Bionano Solve could not be run with optical map alignments supplied as inputs, it was omitted from these comparisons.

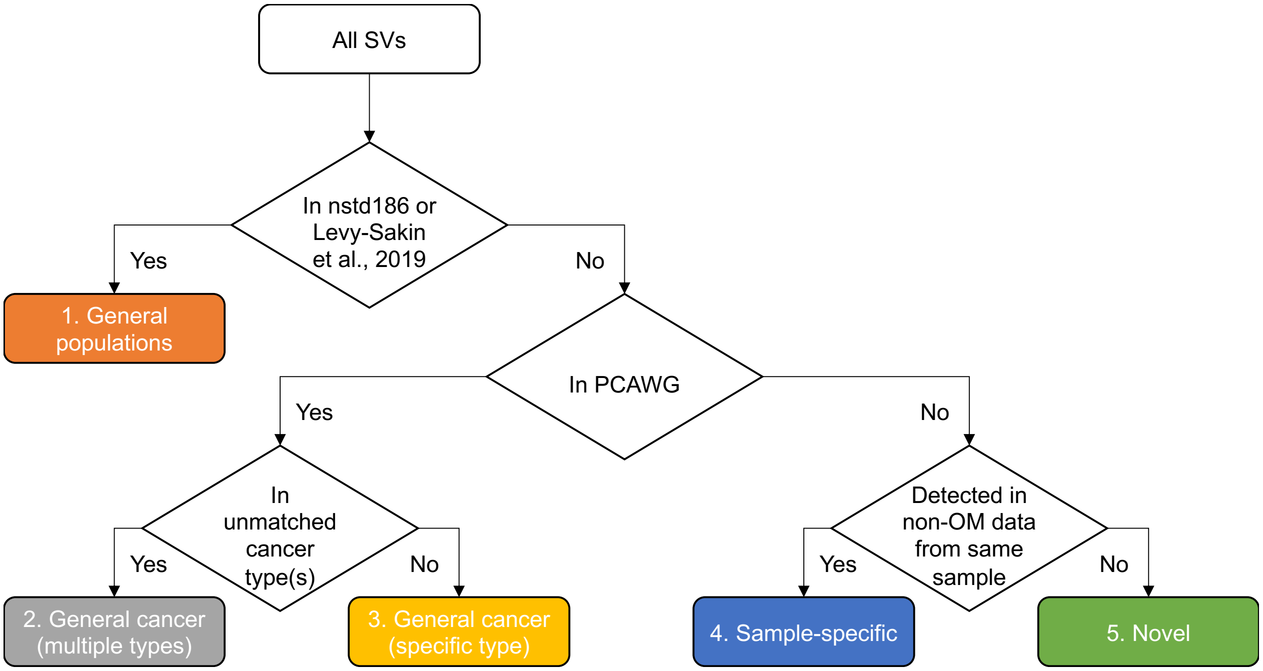

Figure S4: Classification of SVs identified from the real samples based on supports provided by independent data.

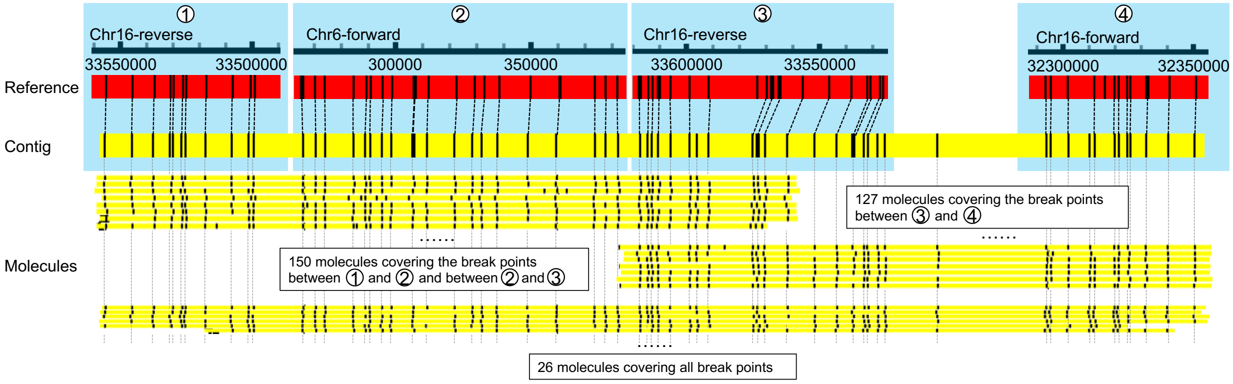

Figure S5: Supporting evidence for a complex SV-containing contig. The alignment between the reference (red bars), optical map contig (thick yellow bar) and individual optical map molecules (thin yellow bars) are shown for the region highlighted in a box with dashed-line boundaries in Figure 6d. Aligned segments (1-4) are numbered in the same way as in Figure 6d.

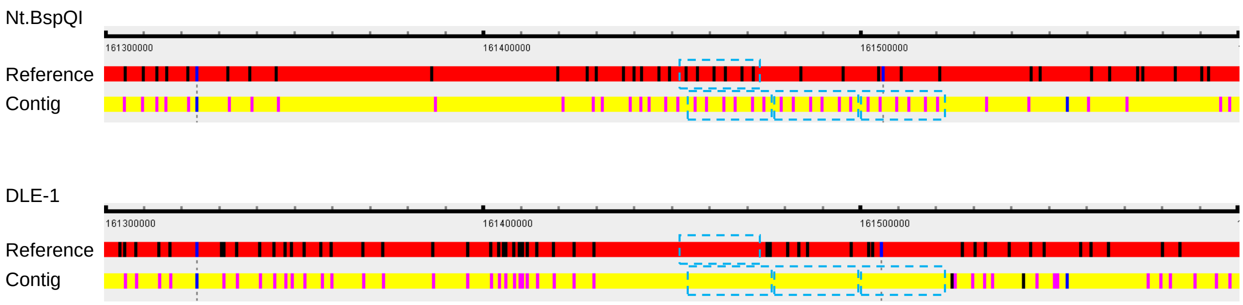

Figure S6: A duplication identified from Nt.BspQI data but not DLE-1 data. The duplication, identified from LNCaP, displays a pattern (highlighted by the blue boxes) that repeats three times in Nt.BspQI data. DLE-1 labels are completely missing in this region.

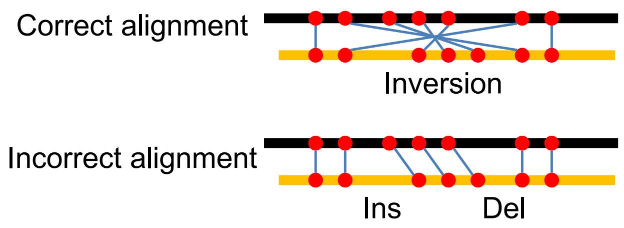

Figure S7: Illustration of a situation that leads to missed call of inversions. The upper part show the correct alignment of the labels on a molecule (yellow bar) with those on a reference (black bar), which should support an inversion. However, due to the symmetric pattern of the labels in the inverted region, the actual alignment produced can be the one shown in the lower part, which instead supports an insertion and a deletion.

S2.3 Supplementary files

Supplementary File 1: List of SVs identified from individual human samples

Supplementary File 2: Unified list of non-redundant novel SVs identified from the different human samples

Supplementary File 3: Lists of genomic regions excluded from SV calling

References

[1] Alden King-Yung Leung, Tsz-Piu Kwok, Raymond Wan, Ming Xiao, Pui-Yan Kwok, Kevin Y. Yip, and Ting-Fung Chan. OMBlast: Alignment tool for optical mapping using a seed-and-extend approach. *Bioinformatics*, 33(3):311–319, 2017.

[2] Alden King-Yung Leung, Nana Jin, Kevin Y. Yip, and Ting-Fung Chan. OMTools: A software package for visualizing and processing optical mapping data. *Bioinformatics*, 33(18):2933–2935, 2017.

[3] Hongzhi Cao, Alex R Hastie, Dandan Cao, Ernest T Lam, Yuhui Sun, Haodong Huang, Xiao Liu, Liya Lin, Warren Andrews, Saki Chan, Shujia Huang, Xin Tong, Michael Requa, Thomas Anantharaman, Anders Krogh, Huanming Yang, Han Cao, and Xun Xu. Rapid detection of structural variation in a human genome using nanochannel-based genome mapping technology. *GigaScience*, 3(1):34, 2014.

[4] Michal Levy-Sakin, Steven Pastor, Yulia Mostovoy, Le Li, Alden KY Leung, Jennifer McCaffrey, Eleanor Young, Ernest T Lam, Alex R Hastie, Karen HY Wong, Claire YL Chung, Walfred Ma, Justin Sibert, Ramakrishnan Rajagopalan, Nana Jin, Eugene YC Chow, Catherine Chu, Annie Poon, Chin Lin, Ahmed Naguib, Wei-Ping Wang, Han Cao, Ting-Fung Chan, Kevin Y Yip, Ming Xiao, and Pui-Yan Kwok. Genome maps across 26 human populations reveal population-specific patterns of structural variation. *Nature Communications*, 10(1):1025, 2019.

[5] Le Li, Alden King-Yung Leung, Tsz-Piu Kwok, Yvonne Y. Y. Lai, Iris K. Pang, Grace Tin-Yun Chung, Angel C. Y. Mak, Annie Poon, Catherine Chu, Menglu Li, Jacob J. K. Wu, Ernest T. Lam, Han Cao, Chin Lin, Justin Sibert, Siu-Ming Yiu, Ming Xiao, Kwok-Wai Lo, Pui-Yan Kwok, Ting-Fung Chan, and Kevin Y Yip. OMSV enables accurate and comprehensive identification of large structural variations from nanochannel-based single-molecule optical maps. *Genome Biology*, 18(1):230, 2017.

[6] Xuesong Hu, Jianying Yuan, Yujian Shi, Jianliang Lu, Binghang Liu, Zhenyu Li, Yanxiang Chen, Desheng Mu, Hao Zhang, Nan Li, Zhen Yue, Fan Bai, Heng Li, and Wei Fan. pIRS: Profile-based illumina pair-end reads simulator. *Bioinformatics*, 28(11):1533–1535, 2012.

[7] Jens Luebeck, Ceyda Coruh, Siavash R Dehkordi, Joshua T Lange, Kristen M Turner, Viraj Deshpande, Dave A Pai, Chao Zhang, Utkrisht Rajkumar, Julie A Law, Paul S Mischel, and Vineet Bafna. AmpliconReconstructor integrates NGS and optical mapping to resolve the complex structures of focal amplifications. *Nature Communications*, 11(1):4374, 2020.

[8] Jesse R. Dixon, Jie Xu, Vishnu Dileep, Ye Zhan, Fan Song, Victoria T. Le, Galip Gürkan Yardımcı, Abhijit Chakraborty, Darrin V. Bann, Yanli Wang, Royden Clark, Lijun Zhang, Hongbo Yang, Tingting Liu, Sriranga Iyyanki, Lin An, Christopher Pool, Takayo Sasaki, Juan Carlos Rivera-Mulia, Hakan Ozadam, Bryan R. Lajoie, Rajinder Kaul, Michael Buckley, Kristen Lee, Morgan Diegel, Dubravka Pezic, Christina Ernst, Suzana Hadjur, Duncan T. Odom, John A. Stamatoyannopoulos, James R. Broach, Ross C. Hardison, Ferhat Ay, William Stafford Noble, Job Dekker, David M. Gilbert, and Feng Yue. Integrative detection and analysis of structural variation in cancer genomes. *Nature Genetics*, 50(10):1388–1398, 2018.

[9] David Y Goldrich, Brandon LaBarge, Scott Chartrand, Lijun Zhang, Henry B Sadowski, Yang Zhang, Khoa Pham, Hannah Way, Chi-Yu Jill Lai, Andy Wing Chun Pang, Benjamin Clifford, Alex R Hastie, Mark Oldakowski, David Goldenberg, and James R Broach. Identification of somatic structural variants in solid tumors by optical genome mapping. *Journal of Personalized Medicine*, 11(2):142, 2021.

[10] Adam Frankish, Sílvia Carbonell-Sala, Mark Diekhans, Irwin Jungreis, Jane E Loveland, Jonathan M Mudge, Cristina Sisu, James C Wright, Carme Arnan, If Barnes, Abhimanyu Banerjee, Ruth Bennett, Andrew Berry, Alexandra Bignell, Carles Boix, Ferriol Calvet, Daniel Cerdán-Vélez, Fiona Cunningham, Claire Davidson, Sarah Donaldson, Cagatay Dursun, Reham Fatima, Stefano Giorgetti, Carlos Garcıa Giron, Jose Manuel Gonzalez, Matthew Hardy, Peter W Harrison, Thibaut Hourlier, Zoe Hollis, Toby Hunt, Benjamin James, Yunzhe Jiang, Rory Johnson, Mike Kay, Julien Lagarde, Fergal J Martin, Laura Martínez Gómez, Surag Nair, Pengyu Ni, Fernando Pozo, Vivek Ramalingam, Magali Ruffier, Bianca M Schmitt, Jacob M Schreiber, Emily Steed, Marie-Marthe Suner, Dulika Sumathipala, Irina Sycheva, Barbara Uszczynska-Ratajczak, Elizabeth Wass, Yucheng T Yang, Andrew Yates, Zahoor Zafrulla, Jyoti S Choudhary, Mark Gerstein, Roderic Guigo, Tim J P Hubbard, Manolis Kellis, Anshul Kundaje, Benedict Paten, Michael L Tress, and Paul Flicek. GENCODE: Reference annotation for the human and mouse genomes in 2023. *Nucleic Acids Research*, 51(D1):D942–D949, 2023.

[11] Ilkka Lappalainen, John Lopez, Lisa Skipper, Timothy Hefferon, J Dylan Spalding, John Garner, Chao Chen, Michael Maguire, Matt Corbett, George Zhou, Justin Paschall, Victor Ananiev, Paul Flicek, and Deanna M Church. DbVar and DGVa: Public archives for genomic structural variation. *Nucleic Acids Research*, 41(D1):D936–D941, 2012.

[12] The ICGC/TCGA Pan-Cancer Analysis of Whole Genomes Consortium. Pan-cancer analysis of whole genomes. *Nature*, 578(7793):82–93, 2020.

[13] Maria Nattestad, Sara Goodwin, Karen Ng, Timour Baslan, Fritz J Sedlazeck, Philipp Rescheneder, Tyler Garvin, Han Fang, James Gurtowski, Elizabeth Hutton, Elizabeth Tseng, Chen-Shan Chin, Timothy Beck, Yogi Sundaravadanam, Melissa Kramer, Eric Antoniou, John D McPherson, James Hicks, W Richard McCombie, and Michael C Schatz. Complex rearrangements and oncogene amplifications revealed by long-read DNA and RNA sequencing of a breast cancer cell line. *Genome Research*, 28(8):1126–1135, 2018.
